# Predicting place of delivery choice among childbearing women in East Africa: a comparative analysis of advanced machine learning techniques

**DOI:** 10.3389/fpubh.2024.1439320

**Published:** 2024-11-27

**Authors:** Habtamu Setegn Ngusie, Getanew Aschalew Tesfa, Asefa Adimasu Taddese, Ermias Bekele Enyew, Tilahun Dessie Alene, Gebremeskel Kibret Abebe, Agmasie Damtew Walle, Alemu Birara Zemariam

**Affiliations:** ^1^Department of Health Informatics, School of Public Health, College of Medicine and Health Sciences, Woldia University, Woldia, Ethiopia; ^2^School of Public Health, College of Medicine and Health Science, Dilla University, Dilla, Ethiopia; ^3^Department of Sport, Physical Education and Health (SPEH), Academy of Wellness and Human Development, Faculty of Arts and Social Sciences, Hong Kong Baptist University, Kowloon, Hong Kong SAR, China; ^4^Department of Health Informatics, College of Medicine and Health Science, Wollo University, Dessie, Ethiopia; ^5^Department of Pediatric and Child Health, School of Medicine, College of Medicine and Health Science, Wollo University, Dessie, Ethiopia; ^6^Department of Emergency and Critical Care Nursing, School of Nursing, College of Medicine and Health Sciences, Woldia University, Woldia, Ethiopia; ^7^Department of Health Informatics, College of Medicine and Health Science, Debre Berhan University, Debre Berhan, Ethiopia; ^8^Department of Pediatrics and Child Health Nursing, School of Nursing, College of Medicine and Health Science, Woldia University, Woldia, Ethiopia

**Keywords:** association rule mining, feature relevance, health facility delivery, home delivery, machine learning algorithms

## Abstract

**Background:**

Sub-Saharan Africa faces high neonatal and maternal mortality rates due to limited access to skilled healthcare during delivery. This study aims to improve the classification of health facilities and home deliveries using advanced machine learning techniques and to explore factors influencing women's choices of delivery locations in East Africa.

**Method:**

The study focused on 86,009 childbearing women in East Africa. A comparative analysis of 12 advanced machine learning algorithms was conducted, utilizing various data balancing techniques and hyperparameter optimization methods to enhance model performance.

**Result:**

The prevalence of health facility delivery in East Africa was found to be 83.71%. The findings showed that the support vector machine (SVM) algorithm and CatBoost performed best in predicting the place of delivery, in which both of those algorithms scored an accuracy of 95% and an AUC of 0.98 after optimized with Bayesian optimization tuning and insignificant difference between them in all comprehensive analysis of metrics performance. Factors associated with facility-based deliveries were identified using association rule mining, including parental education levels, timing of initial antenatal care (ANC) check-ups, wealth status, marital status, mobile phone ownership, religious affiliation, media accessibility, and birth order.

**Conclusion:**

This study underscores the vital role of machine learning algorithms in predicting health facility deliveries. A slight decline in facility deliveries from previous reports highlights the urgent need for targeted interventions to meet Sustainable Development Goals (SDGs), particularly in maternal health. The study recommends promoting facility-based deliveries. These include raising awareness about skilled birth attendance, encouraging early ANC check-up, addressing financial barriers through targeted support programs, implementing culturally sensitive interventions, utilizing media campaigns, and mobile health initiatives. Design specific interventions tailored to the birth order of the child, recognizing that mothers may have different informational needs depending on whether it is their first or subsequent delivery. Furthermore, we recommended researchers to explore a variety of techniques and validate findings using more recent data.

## Introduction

Ensuring universal access to high-quality healthcare services, particularly for maternal and child health, is a crucial global goal rooted in the principles of primary healthcare (1, 2). Within the realm of maternal health, this objective involves providing comprehensive and easily accessible healthcare services tailored specifically to women of reproductive age. These services encompass essential components such as ANC, health facility delivery, and postnatal care (3, 4).

By placing a strong emphasis on primary healthcare, we can significantly enhance the wellbeing of both mothers and children through the provision of essential, accessible, and affordable healthcare services (3, 4). However, despite these intentions, challenges remain in the provision of maternal healthcare services, especially in developing regions. Notably, limited access to health facility delivery and inadequate ANC visits contribute to suboptimal health outcomes in East Africa (5–8).

Extensive research has established a clear link between health facility delivery and maternal and neonatal mortality rates (9, 10). In particular, home deliveries, which often lack access to skilled healthcare professionals and emergency obstetric care, expose mothers and newborns to increased risks of adverse outcomes (11, 12). Complications during childbirth such as postpartum hemorrhage, obstructed labor, infections, birth asphyxia, and neonatal sepsis pose substantial threats to the health and survival of both mothers and infants (13, 14). Furthermore, the absence of immediate interventions in home settings can lead to delays in recognizing and managing these complications, thereby exacerbating their severity and contributing to poorer health outcomes (15).

Given that childbirth complications are a leading cause of maternal and neonatal mortality, delivering in health facilities emerges as a prominent solution. The World Health Organization (WHO) identifies severe bleeding (mainly postpartum), infections (typically after delivery), high blood pressure during pregnancy (pre-eclampsia and eclampsia), complications from delivery, and unsafe abortions as responsible for 75% of maternal deaths (16). For instance, severe postpartum hemorrhage alone accounts for 27.1% of maternal fatalities globally, while obstructed labor contributes 17.9% (17).

Moreover, postpartum infections are a major cause of maternal death, with ~5 million cases of pregnancy-related infections reported annually, resulting in around 75,000 deaths (18). Additionally, gestational diabetes affects about 20 million births each year (19), and hypertensive disorders in pregnancy lead to ~76,000 maternal and 500,000 prenatal deaths globally each year (20). Epidemiological studies further report that the prevalence of hypertensive disorders in pregnancy ranges from 5.2 to 8.2% for gestational hypertension and from 0.2 to 9.2% for preeclampsia (21).

As of 2023, about 1.2 million pregnant women worldwide were living with HIV (22), with mother-to-child transmission accounting for 9% of new infections (23). For neonates, premature births, birth complications (such as birth asphyxia and trauma), neonatal infections, and congenital anomalies collectively account for nearly 40% of deaths in children under five (24). Therefore, delivering in health facilities is one of the safest methods to manage these complications and mitigate their severity, ultimately protecting the lives of both mothers and their newborns. Access to skilled healthcare professionals and emergency obstetric care in health facilities significantly reduces the risks associated with childbirth complications, underscoring the vital role of health facility delivery in improving maternal and neonatal health outcomes.

In the context of Sub-Saharan Africa, particularly alarming rates of neonatal and maternal mortality persist due to inadequate health facility delivery. For example, in 2017, the region reported a maternal mortality ratio of 542 per 100,000 live births, highlighting the urgent need for intervention (7, 8). Additionally, in 2018, Sub-Saharan Africa recorded the highest neonatal mortality rate among regions defined by the SDGs, with 28 deaths occurring per 1,000 live births (25, 26).

To address these pressing challenges, the WHO, in collaboration with governments and partners, has implemented various initiatives in Sub-Saharan Africa. These initiatives focus on developing and implementing comprehensive maternal and child health programs, promoting community-based interventions, enhancing emergency obstetric care services, and encouraging skilled birth attendance (27, 28). Despite these efforts, a significant proportion of women in East Africa still choose home births (29, 30).

The choice of delivery location is influenced by a wide array of factors. These factors encompass residence (31), age (31, 32), education level of mothers and husband (33), ANC visit (34), wealth status (34), religion (31), women's occupation (31), husband occupation (32), sex of household head (35), media access (29, 31), the timing of the first ANC check (36, 37), number of pregnancies (38), age at first marriage (39), preceding birth interval (40), distance from a health facility (33, 41), mobile phone ownership (42), and birth order (42, 43).

While previous studies have examined the factors influencing the choice of delivery location using Demographic and Health Survey (DHS) data from various countries (29, 44), a deeper understanding requires the utilization of advanced machine learning algorithms and data science techniques. Such an approach enables the discovery of hidden patterns and relationships that may not be easily discernible through traditional statistical methods.

Consequently, we propose a study aimed at evaluating the potential improvements in classification performance achieved by employing a diverse range of advanced machine learning and data science techniques to distinguish between home and health facility deliveries. Additionally, the study will investigate the key factors that influence the decision-making process among childbearing women in East Africa when choosing between these two delivery options.

## Related works

The topic of health facility vs. home delivery among women of reproductive age has been extensively studied in various contexts (30, 31, 35, 40, 44–57). While traditional statistical methods have been commonly utilized, researchers have recognized their limitations in capturing complex relationships and interactions among multiple influencing factors (58). To address these shortcomings, machine learning techniques have been increasingly applied, yielding promising results in predicting delivery locations and improving health outcomes for mothers and newborns.

For instance, a study conducted in Zanzibar employed logistic regression, LASSO regression, random forest, and artificial neural networks to predict delivery locations, achieving accuracy rates between 68 and 77% (59). Another study in Zanzibar focused on evaluating the vulnerability of algorithms used in community health worker-led maternal health programs, emphasizing the critical need for accurate data monitoring strategies to effectively target high-risk women (60).

In Afghanistan, a web-based predictive model utilizing machine learning algorithms, particularly random forest, achieved an impressive accuracy of 84.23%. This highlights the potential for targeted interventions to enhance the utilization of skilled child delivery services and reduce maternal and child mortality rates (61). Similarly, a study in Ethiopia that explored determinants of skilled delivery service utilization developed a predictive model using the J48 algorithm, demonstrating exceptional accuracy of 98% (62).

Despite these advancements, significant research gaps remain in the exploration and application of advanced machine learning algorithms, data balancing techniques, and tuning methods, particularly when applied to large datasets. Previous studies have often been constrained by their reliance on relatively small datasets, which limits the generalizability of their findings.

To bridge this research gap, our study aims to conduct a comprehensive investigation by leveraging a relatively large dataset. We will explore and experiment with 12 cutting-edge advanced machine learning algorithms, along with various data balancing techniques and tuning methods, to enhance accuracy and precision in distinguishing between health facility and home deliveries.

## Method

### Data source

This study utilized secondary data from the most recent Demographic and Health Surveys (DHS) conducted in 12 East African countries: Ethiopia (2016), Kenya (2022), Uganda (2016), Tanzania (2022), Burundi (2017), Rwanda (2015), Madagascar (2021), Mozambique (2015), Zimbabwe (2015), Zambia (2018), Malawi (2016), and Comoros (2012). For each country, the most recent DHS data was used; if multiple surveys were available, the latest one was taken. The data was obtained from the official DHS Program database (URL: https://dhsprogram.com/data/available-datasets.cfm). Ethical approval was obtained from the Institutional Review Board for the DHS Program to ensure compliance with research guidelines.

The DHS Program has conducted standardized cross-sectional surveys in over 90 countries, gathering comprehensive and representative data on aspects such as population, health, HIV, and nutrition. These surveys employed a multi-stage stratified sampling approach, where participants were selected from households within designated clusters. Sampling strata were created based on urban and rural sectors, and enumeration areas were chosen using probability proportional to size. Within the selected enumeration areas, households were chosen using equal probability systematic sampling (63).

The study specifically targeted childbearing women in East Africa, those between the ages of 15 and 49 who had given birth within the past 5 years. The analysis included a substantial sample size of 86,009 individuals across the 12 countries mentioned. The dataset used in the study consisted of 19 distinct features that were taken into account during the analysis.

### Study variables and measurements

In this study, the variable of interest was health facility delivery, defined as women giving birth in healthcare facilities, including government, private, and non-government health institutions. Reproductive-age women who delivered in these facilities were categorized as “health facility delivery” (coded as 1), while those who gave birth outside of healthcare facilities were categorized as “home delivery” (coded as 0) (63). The study considered several independent variables after reviewing the literature, including place of residence, religion, media exposure, sex of household head, birth order number, birth interval, timing of the first ANC check, number of children in the family, current marital status, ANC visit, working status of the mother, owns the mobile telephone, wealth status, age of household head, husband/partner educational level, age of the mother, education level of mothers, and distance from health facilities.

### Data preprocessing

The first step in machine learning is data pre-processing, which involves preparing and transforming the data to make it understandable for computers (64). In this study, the machine learning process focused on the outcome variable of delivery location, along with various independent variables outlined in the above subsection.

**Figure 1:**
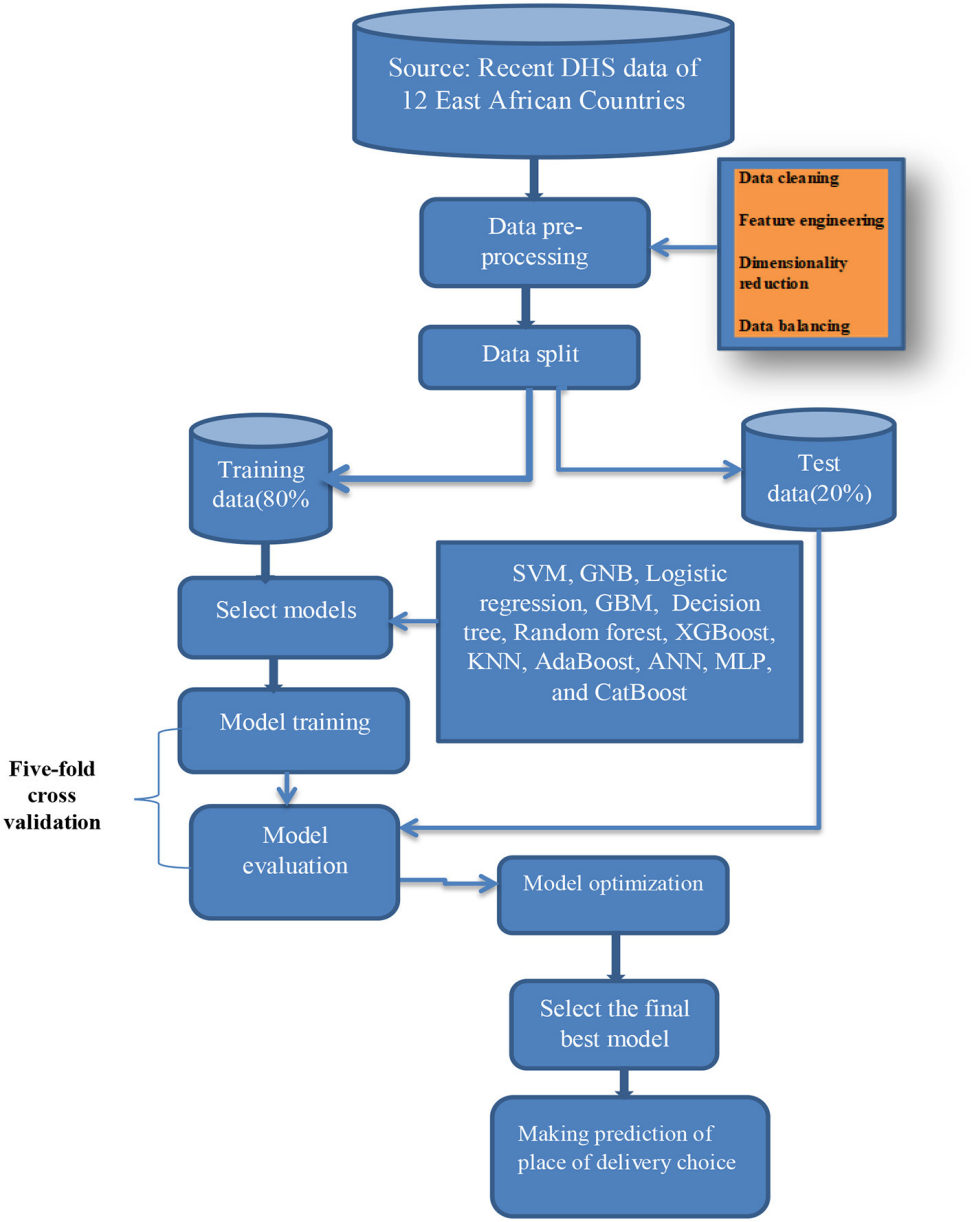
Study work flow diagram. ANN, Artificial Neural Network; GNB, Gaussian Naive Bayes; GBM, Gradient Boosting Machines; KNN, K-Nearest Neighbors; MLP, Multi-Layer Perceptron; SVM, Support Vector Machine; XGBoost, Extreme Gradient Boosting.

Our machine learning workflow involved a continuous improvement process for our models. We selected and engineered features, chose the appropriate model, trained the model, evaluated its performance, optimized its parameters through cross-validation, selected the top-performing final model, and deployed it to predict the place of delivery (65). We refined our models through an iterative approach, continuously making improvements. Figure 1 visually represents the steps in our workflow, but it may not include all the recurring tasks that were executed.

### Data cleaning

During the data analysis process, a comprehensive data cleaning approach was employed to ensure the quality and integrity of the dataset. In this study, no redundant data entries were identified.

The missing rate for all variables in our study was found to be below 10%. To address missing values, the K-Nearest Neighbors (KNN) imputation technique was utilized. KNN imputation is a widely adopted method that leverages information from neighboring data points to impute missing values. KNN was chosen for its ability to utilize surrounding information, handle different data types, preserve structure, and its established reliability in missing value imputation (66, 67).

To identify outliers, we utilized various visualization techniques such as scatter plots, box plots, and histograms. Few outliers were removed based on the recommendation of the DHS guideline (63). Additionally, we assessed multicollinearity by examining the correlation matrix, considering a correlation value exceeding 0.8 between two variables as indicative of high correlation (68, 69). Our analysis confirmed that no multicollinearity was observed among the variables in this study.

**Figure 2:**
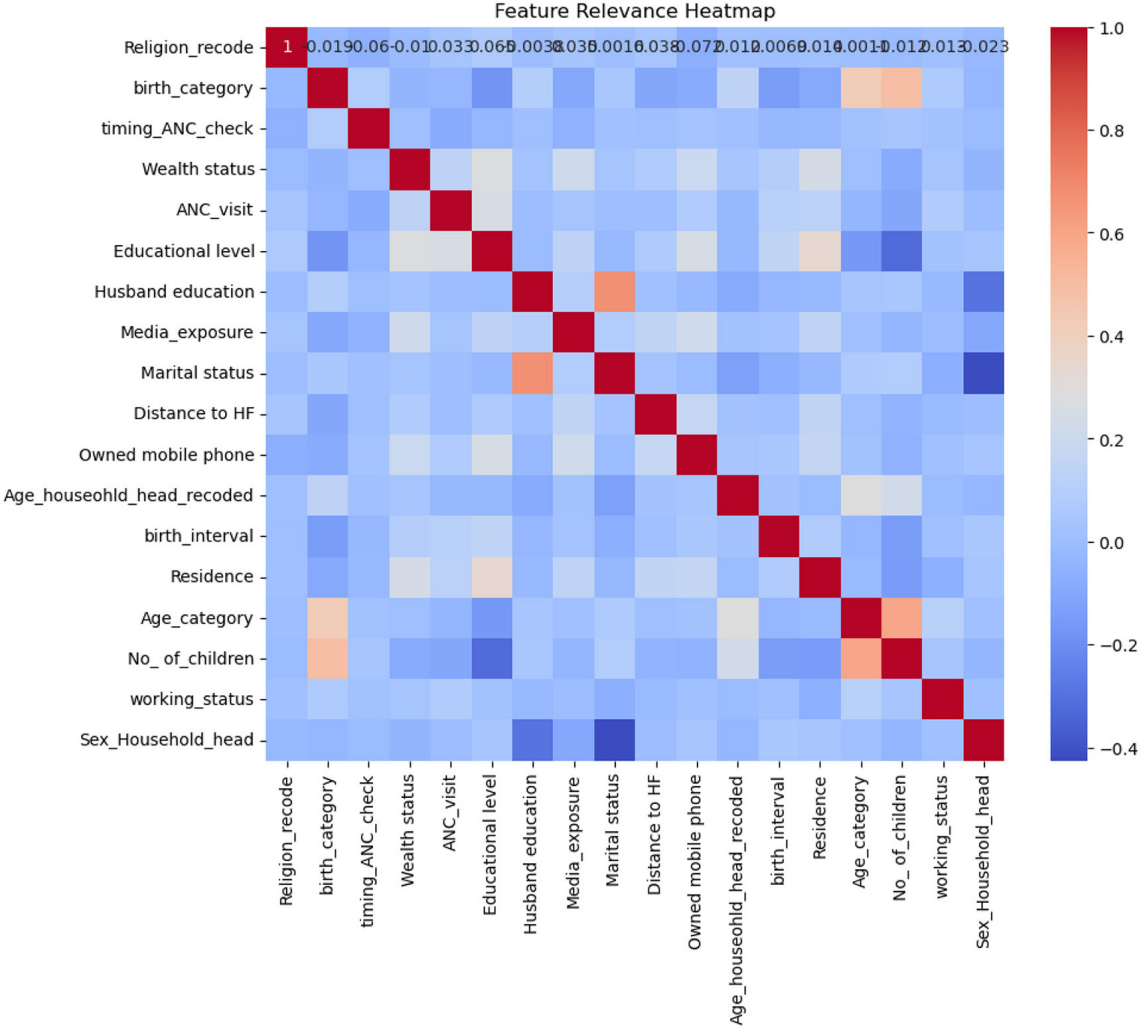
Heat map of the correlation matrix showing feature relevance.

As shown in Figure 2, the highest correlation was observed between marital status and husband's education, and there was also some correlation between the number of children and the mother's age; however, all correlations remained below 0.8. Therefore, no significant multicollinearity was detected among the variables.

### Feature engineering

Feature engineering is the process of selecting, acquiring, and transforming the most relevant features from the available data to build machine learning models that are both precise and efficient (70). In our research, we used one-hot encoding to encode nominal categorical variables and label encoding for ordinal categorical variables (71).

### Dimensionality reduction

In our study, we focused on enhancing model performance and simplifying the dataset through various dimensionality reduction techniques. These techniques included univariate selection, recursive feature elimination (RFE), random forest feature elimination, principal component analysis (PCA), lasso regression, and Boruta-based feature selection (72). After conducting thorough experimentation and comparing the results across different feature selection methods, we determined that the Boruta-based approach stood out as the most effective in terms of accuracy and robustness.

One of the key advantages of the Boruta algorithm is its ability to evaluate feature importance by comparing their performance against randomly generated shadow features. This approach ensures a comprehensive and unbiased assessment of feature significance, allowing only the most informative features to be selected for our predictive model. Moreover, the Boruta algorithm successfully distinguishes true signals from noise by comparing features against shadow features, resulting in a more reliable and robust feature selection process (73, 74).

By incorporating the features selected by the Boruta algorithm, we observed improved accuracy and robustness in predicting the place of delivery. Prediction accuracy was measured using metrics such as accuracy, precision, recall, F1-score, and AUC, while 5-fold cross-validation was employed to assess robustness. This underscores the practical benefits of the Boruta-based feature selection method within the context of our dataset.

**Figure 3:**
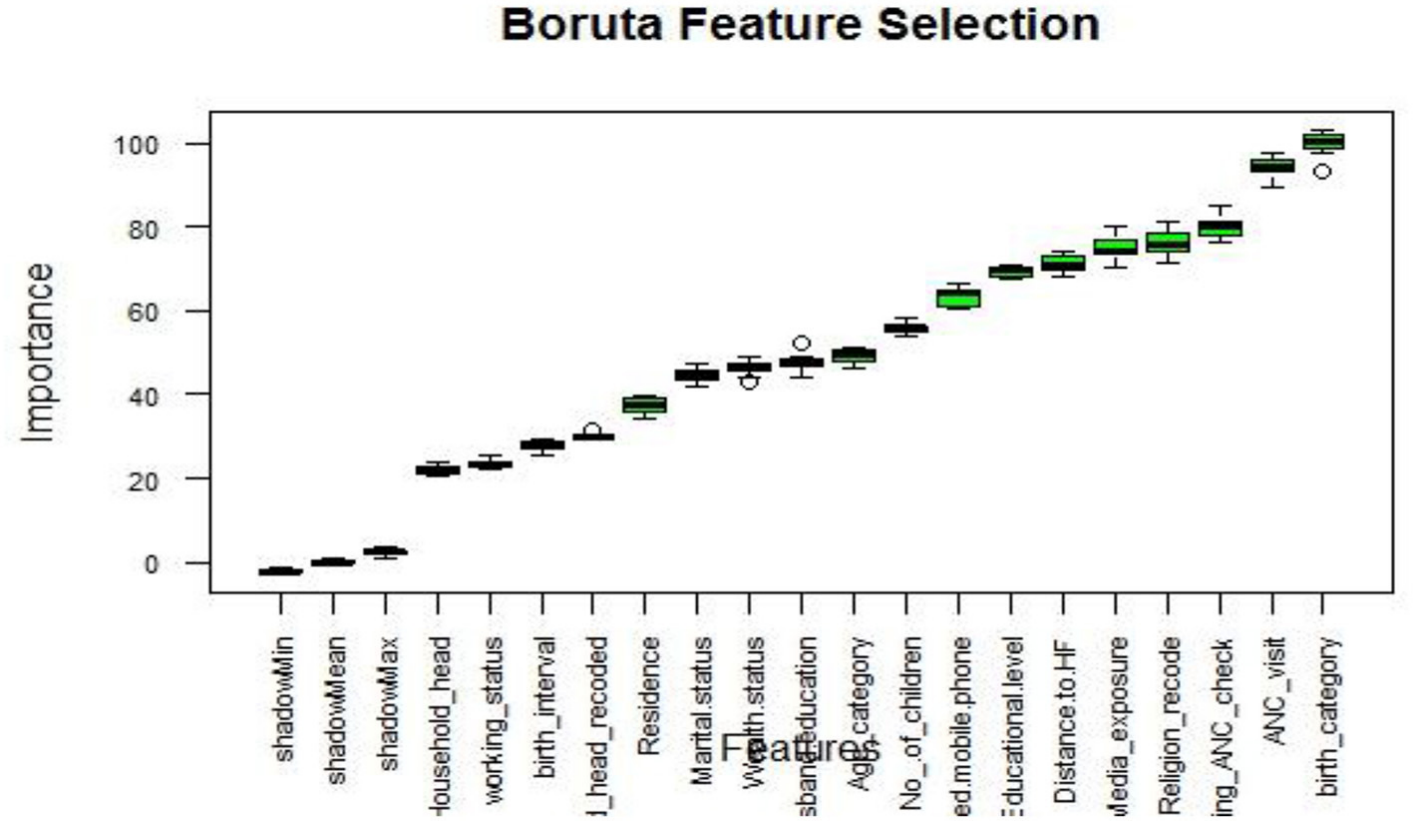
Feature selection using Boruta algorithm.

Boruta algorithm graph visualized the importance of variables, highlighting significant variables in green, unimportant variables in red, and tentative variables in yellow (75). In our comprehensive analysis, the Boruta algorithm graph (Figure 3) showed all variables are important. Consequently, we used all variables to predict the place of delivery and explore data patterns through association rule mining.

### Data balancing

To address the class imbalance, it's helpful to experiment with various data balancing methods and select the most effective one for the specific dataset (76–78). To address the issue of class imbalance in our dataset, which consists of a binary outcome variable and categorical independent variables (with a few of them being binary), we conducted an extensive review of scientific literature (79, 80) and experimented with seven different data balancing methods. These methods were carefully selected based on their appropriateness for our specific dataset. The techniques we employed are as follows: Under-sampling, Over-sampling, Adaptive Synthetic Sampling (ADASYN), Synthetic Minority Oversampling Technique (SMOTE), SMOTE-ENN (Edited Nearest Neighbor), SMOTE-Tomek, and NearMiss algorithm.

By conducting a comprehensive assessment of model performance, and considering various metrics, we compared machine learning algorithms trained on balanced data using these different balancing techniques. This process enabled us to identify the most effective approach for addressing the class imbalance in our dataset. We selected the balancing technique that demonstrated superior performance for further experimentation and final prediction.

In this study, we compared the performance of each machine learning algorithm across different data balancing techniques. The results showed that SMOTE-ENN significantly outperformed the other methods, demonstrating a substantial advantage over them. Consequently, we chose the SMOTE-ENN technique for further analysis and optimization (see Table 1 for more details).

**Table 1:** Comparison of data balancing techniques across each machine learning algorithms.

**Algorithms**	**Performance metrics**	**Unbalanced data**	**Under-sampling**	**Over-sampling**	**ADASYN**	**SMOTE**	**SMOTE-ENN**	**SMOTE-TOMEK**	**Near miss**
SVM	Accuracy	81.0%	69.0%	72.0%	73.0%	75.0%	88.0%	76.0%	76.0%
	AUC	0.71	0.71	0.79	0.81	0.83	0.95	0.84	0.83
GNB	Accuracy	68.0%	64.0%	64.0%	65.0%	67.0%	80.0%	68.0%	67.0%
	AUC	0.71	0.75	0.71	0.74	0.76	0.88	0.77	0.77
Logistic regression	Accuracy	80.0%	68.0%	68.0%	70.0%	72.0%	84.0%	73.0%	71.0%
	AUC	0.74	0.74	0.74	0.78	0.80	0.92	0.81	0.78
Decision tree	Accuracy	72.0%	61.0%	80.0%	73.0%	74.0%	87.0%	75.0%	65.0%
	AUC	0.59	0.61	0.81	0.73	0.75	0.85	0.75	0.66
Random forest	Accuracy	81.0%	68.0%	85.0%	80.0%	81.0%	92.0%	82.0%	73.0%
	AUC	0.74	0.75	0.94	0.88	0.89	0.98	0.90	0.81
GBM	Accuracy	81.0%	69.0%	69.0%	72.0%	73.0%	86.0%	74.0%	76.0%
	AUC	0.76	0.76	0.77	0.80	0.82	0.93	0.82	0.83
XGBoost	Accuracy	81.0%	68.0%	73.0%	75.0%	76.0%	90.0%	77.0%	76.0%
	AUC	0.76	0.75	0.81	0.83	0.84	0.96	0.85	0.84
KNN	Accuracy	81.0%	67.0%	69.0%	72.0%	75.0%	88.0%	76.0%	69.0%
	AUC	0.72	0.73	0.77	0.81	0.83	0.95	0.84	0.77
MLP	Accuracy	76.0%	62.0%	73.0%	72.0%	73.0%	89.0%	74.0%	70.0%
	AUC	0.68	0.67	0.79	0.79	0.81	0.94	0.82	0.77
AdaBoost	Accuracy	81.0%	68.0%	68.0%	71.0%	72.0%	85.0%	73.0%	73.0%
	AUC	0.75	0.75	0.75	0.79	0.80	0.92	0.82	0.81
CatBoost	Accuracy	82.0%	69.0%	73.0%	75.0%	76.0%	89.0%	77.0%	77.0%
	AUC	0.77	0.77	0.81	0.83	0.85	0.96	0.86	0.85
ANN	Accuracy	76.0%	63.0%	73.0%	75.0%	74.0%	88.0%	75.0%	71.0%
	AUC	0.69	0.68	0.79	0.80	0.82	0.94	0.83	0.78

AUC, Area under the Receiver Operating Characteristic Curve; ADASYN, Adaptive Synthetic Sampling; ANN, Artificial Neural Network; ENN, Edited Nearest Neighbor; GNB, Gaussian Naive Bayes; GBM, Gradient Boosting Machines; KNN, K-Nearest Neighbors; MLP, Multi-Layer Perceptron; SMOTE, Synthetic Minority Oversampling Technique; SVM, Support Vector Machine; XGBoost, Extreme Gradient Boosting.

Additionally, Supplementary material 1 provides a detailed graphical comparison of the performance of machine learning algorithms across various data balancing techniques and in the context of imbalanced data.

### Model selection and development

In our research, we focused on predicting the place of delivery. We aimed to classify the place of delivery into two groups: “health facility delivery” and “home delivery.” To ensure accurate predictions, we needed to select suitable classifiers capable of effectively handling this classification task.

To accomplish this, we used the scikit-learn version 1.3.2 packages in Python, implemented within Jupyter Notebook. We employed 12 advanced machine learning algorithms to evaluate their predictive capabilities in distinguishing the place of delivery. The selection of these algorithms was based on their suitability for classification tasks and the nature of our dataset (81–83).

The algorithms we utilized include SVM with kernel methods, Gaussian Naive Bayes (GNB), logistic regression, decision tree, random forest, gradient boosting machines (GBM), eXtreme Gradient Boosting (XGBoost), AdaBoost, KNN algorithm, CatBoost Classifier, multilayer perceptron (MLP) neural network, and artificial neural networks (ANN) using TensorFlow. By incorporating a diverse set of algorithms, we aimed to explore different modeling approaches and assess their effectiveness in predicting the place of delivery (82).

### Model training, evaluation, and optimization

In our study, we employed a straightforward method of dividing the data into two sets: an 80% (68,807 cases) training set and a 20% (17,202 cases) testing set. To assess the performance of each predictive model, we used various measurements, such as accuracy, precision, recall/sensitivity, F1-score, specificity, and AUC. Using these metrics, we conducted a comprehensive evaluation of each predictive model, considering overall correctness, accurate positive predictions, identification of positive instances, balance, and discriminatory ability (84).

In addition, we conducted a comprehensive analysis of the hyperparameters to optimize and enhance the model's performance. During model optimization, we systematically explored grid search, random search, and Bayesian optimization to identify the optimal hyperparameter settings. We compared the results from these techniques to determine which configurations yielded the highest performance. It is recommended to experiment with different tuning techniques and select the one that demonstrates superior performance (85, 86). To ensure robust performance evaluation, we employed cross-validation techniques and compared different options such as 3-, 5-, and 10-fold validations. Upon analysis, we found that 5-fold cross-validation provided the best results for our specific dataset. Therefore, we utilized the 5-fold cross-validation approach to ensure reliable and accurate performance evaluation (87).

To improve the model's accuracy and reliability, we carried out model calibration. By fine-tuning the model through calibration, we enhanced its predictive capabilities, resulting in more precise forecasts of the desired outcome (88).

Moreover, we compared different kernel methods specifically for the SVM model. We experimented with five commonly used types of kernel methods: linear kernel, polynomial kernel, radial basis function (RBF) kernel, sigmoid kernel, and Gaussian kernel (89). After conducting evaluations, we determined that the polynomial kernel method exhibited the highest performance for SVM, and thus we selected and employed it. Our objective was to select the kernel function that produced the best results for the SVM model through a thorough evaluation and comparison process.

### Model interpretability

To enhance the interpretability of our model, we utilized association rule mining techniques to uncover hidden patterns and relationships within the dataset. This involved applying the widely adopted Apriori algorithm, specifically designed for association rule mining. Through this algorithm, we identified frequent item sets and extracted meaningful association rules using measures such as lift and confidence. The lift measure quantified the strength of associations between variables, revealing the influence of one variable on the occurrence of another. Confidence, on the other hand, indicated the reliability of association rules by showing how often the consequent variable appeared when the antecedent variable was present (90–92).

Furthermore, we employed the final top-performing machine learning model to select relevant features for prediction. This process allowed us to evaluate the importance of different features and choose those that had the greatest impact on the model's performance. By incorporating feature relevance selection, we improved the interpretability of our model, emphasizing the influential variables in making predictions.

## Results

### Descriptive results of the background characteristics

The study extensively analyzed the descriptive and socio-demographic characteristics of 86,009 women of reproductive age. Among the participants, the highest percentage, which represented 39,639 individuals (46.09%), were between the ages of 25 and 34. In terms of their place of residence, the majority of the study participants, comprising 64,548 individuals (75.1%), came from rural areas. For more detailed information, please refer to Table 2.

**Table 2:** Individual characteristics of reproductive age group women in East African countries (*n* = 86,009).

**Variable**	**Category**	**Frequency (*n*)**	**Percent (%)**
Residence	Urban	21,461	24.90%
	Rule	64,548	75.10%
Religion	Catholic	24,345	28.31%
	Protestant	24,361	28.32%
	Muslim	7,462	8.68%
	Adventist	5,649	6.57%
	Jehovah	17,301	20.12%
	Tradition animist	4,166	4.84%
	No religion	713	0.83%
	Sect	361	0.42%
	Other	1,651	1.92%
Educational status	No education	19,191	22.31%
	Primary education	41,242	47.95%
	Secondary education	21,540	25.05%
	Higher education	4,036	4.69%
Age (in years)	15–24	26,570	30.89%
	25–34	39,639	46.09%
	35–49	19,800	23.02%
Marital status	Divorced	2,687	3.12%
	never in union	5,374	6.25%
	Widowed	1,458	1.70%
	No longer living together	4,996	5.81%
	living with partner	12,963	15.07%
	Married	58,531	68.05%
Timing of first ANC check	During first trimester	31,789	36.96%
	During second trimester	52,878	61.48%
	During third trimester	1,342	1.56%
Owns mobile telephone	Yes	30,939	35.97%
	No	55,070	64.03%
Wealth index	Poorest	21,058	24.48%
	Poorer	17,015	19.78%
	Middle	16,123	18.75%
	Richer	15,913	18.50%
	Richest	15,900	18.49%
Number of children	≤ 4	54,077	62.87%
	>4	31,932	37.13%
Working status	Have work	59,540	69.23%
	Have no Work	26,469	30.77%
Media exposure	Yes	44,840	52.13%
	No	41,169	47.87%
ANC visit	No visit	5,612	6.52%
	2–3 visit	35,605	41.40%
	4 and above visit	44,792	52.08%
Short birth interval	Long birth	4,517	5.25%
	Normal birth	22,293	25.92%
	Short birth	59,199	68.83%
Distance to health facility	Big problem	31,977	37.18%
	Not big problem	54,032	62.82%
Birth order category	1	18,940	22.02%
	2–3	32,183	37.42%
	4–5	18,950	22.03%
	6 and above	15,936	18.53%
Husband educational level	No education	17,503	20.35%
	Primary	38,575	44.85%
	Secondary	23,059	26.81%
	Higher	6,872	7.99%
Sex of house hold head	Female	21,486	24.98%
	Male	64,523	75.02%
Age of household head	15–19	488	0.57%
	20–29	21,714	25.25%
	30–49	49,373	57.40%
	50 and above	14,434	16.78%

### Prevalence of health facility delivery in East Africa

**Figure 4:**
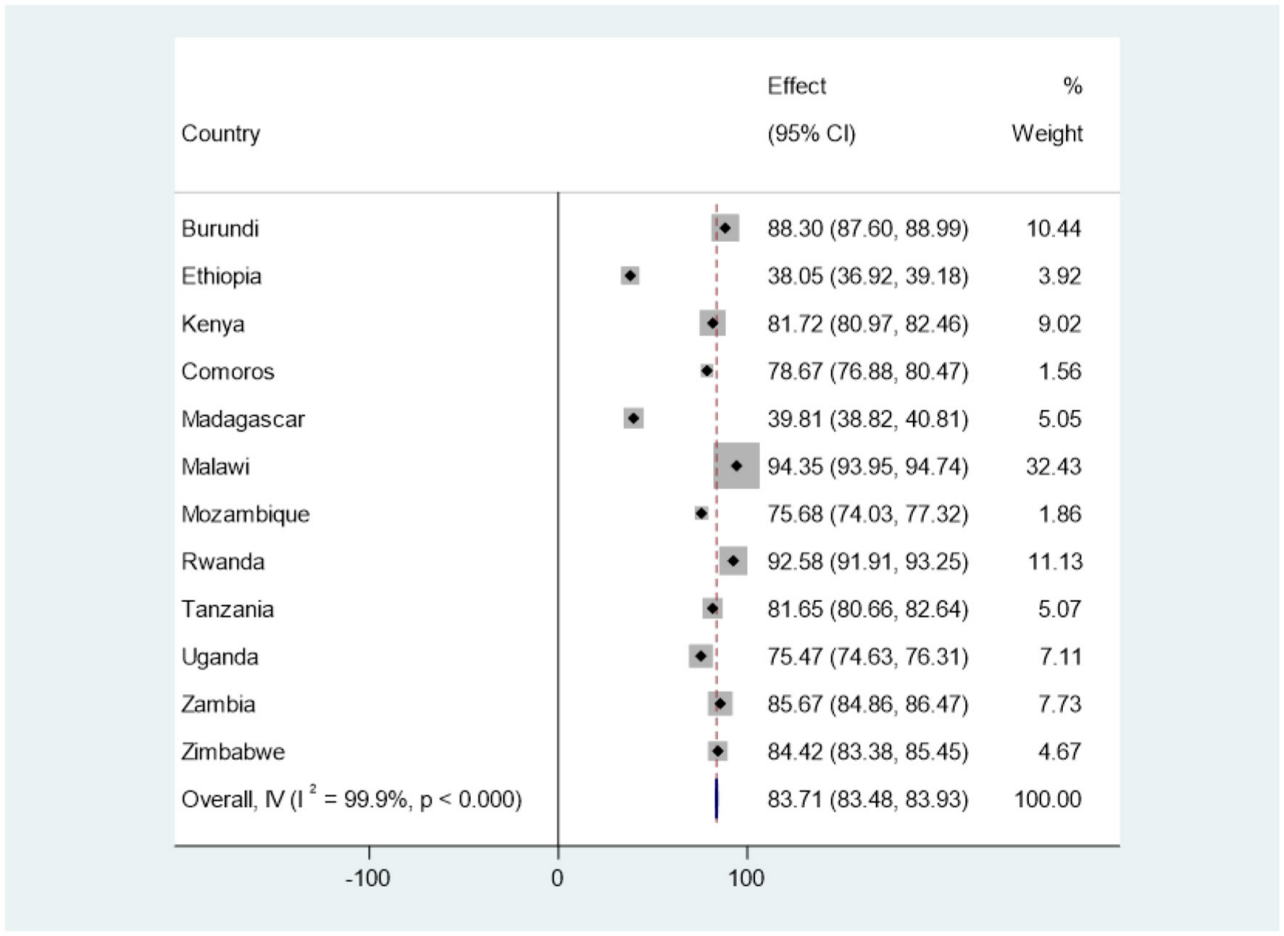
The prevalence of health facility delivery among reproductive age women in East Africa countries using forest tree plot.

Based on the analysis of the recent DHS dataset in our study, as described in the methodology section, it was revealed that the overall prevalence of health facility delivery among women of reproductive age in East Africa was 83.71% (95% CI: 83.48, 83.93). Notably, Ethiopia had the lowest rate of health facility delivery, with only 38.05% (95% CI: 36.92, 39.18) of women accessing such services. Conversely, Malawi had the highest prevalence of health facility delivery, with 94.35% (95% CI: 93.95, 94.74) of women delivering at a health facility. For a more comprehensive breakdown of health facility-based deliveries in each country, please refer to Figure 4.

### Machine learning analysis of the place of delivery

In this subsection, we present the performance of each machine learning algorithm both before and after optimization.

#### Comparative analysis of machine learning models using balanced data

**Figure 5:**
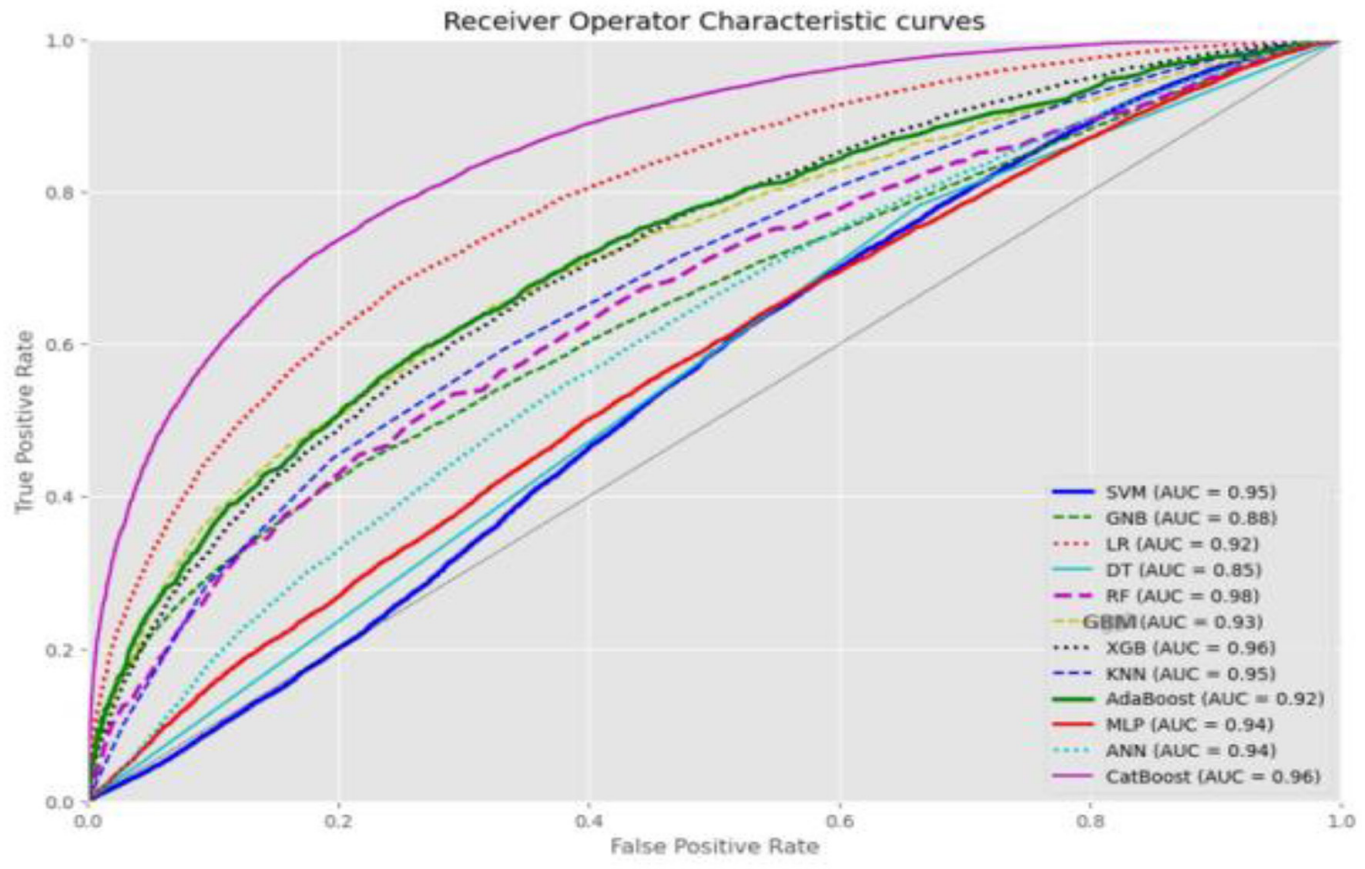
ROC curve value of each machine learning algorithm using balanced data.

As outlined in the methodology, we selected SMOTE-ENN for further analysis and optimization due to its significant effectiveness. The analysis of the balanced data using SMOTE-ENN revealed that Random Forest was the top-performing algorithm, followed by CatBoost and XGBoost. The ROC curve value for SMOTE-ENN is presented in Figure 5. For detailed performance metrics, please refer to Supplementary material 1.

### Performance comparisons of optimized machine learning models

We compared the performance of 12 machine learning algorithms for predicting the place of delivery using three different tuning techniques. We employed techniques such as grid search, random search, and Bayesian optimization for tuning the model. The results demonstrated excellent performance across all 12 algorithms, although performance varied depending on the tuning technique.

In the grid search technique, the random forest algorithm achieved the highest performance metrics, with an AUC of 0.98 and an accuracy of 92.0%. The KNN and GBM closely followed with accuracies of 92.0 and 91.0%, respectively, and corresponding AUC values of 0.96 and 0.97. Most algorithms achieved an AUC above 0.90, except for GNB and decision trees, which had AUC values of 0.88 and 0.86, respectively.

In the random search technique, the random forest algorithm demonstrated superior performance metrics, with an AUC of 0.98 and an accuracy of 93.0%. The XGBoost and CatBoost algorithms closely followed with accuracies of 92.0% and AUC values of 0.97. Similar to the grid search results, most algorithms achieved an AUC above 0.90, except for GNB and decision trees, which had AUC values of 0.88.

Using the Bayesian optimization technique, the SVM and CatBoost algorithms showcased the best performance metrics, in which both of those algorithms scored an accuracy of 95% and AUC of 0.98. The KNN and MLP algorithms closely followed, achieving accuracies of 90.0% with respective AUC values of 0.96 and 0.95. Most algorithms achieved an AUC above 0.90, except for GNB and GBM, which had AUC values of 0.88 and 0.84, respectively.

Overall, the comprehensive evaluation demonstrated excellent performance across all 12 machine learning algorithms, with consistent and comparable results. Different tuning techniques yielded the best outcomes for different algorithms, with random search, grid search, and Bayesian optimization showing notable performance in specific cases.

**Figure 6:**
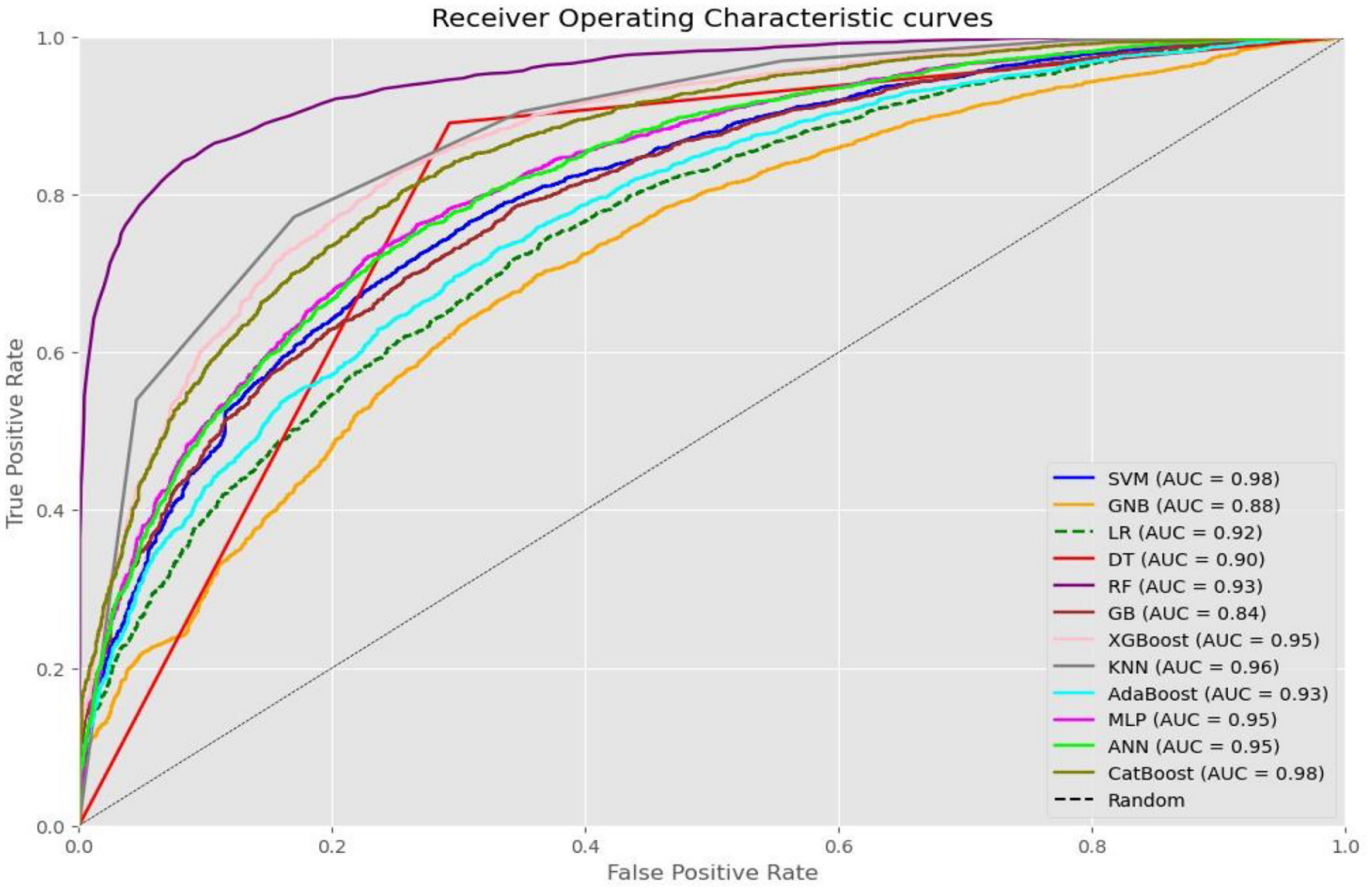
A ROC curve value of each machine learning algorithm after optimized using Baysian optimization technique.

The top-performing algorithms were obtained using Bayesian optimization tuning. Although variations in performance were observed, no single technique consistently outperformed all aspects of the machine learning algorithms. The top-performing algorithms across all metrics were SVM and CatBoost with Bayesian optimization tuning, both scoring an accuracy of 95%, an AUC of 0.98, and showing insignificant differences in comprehensive analysis of metrics performance such as accuracy, precision, recall/sensitivity, F1-score, specificity, and AUC (see Figure 6).

To get a detailed comparison of the 12 machine learning algorithms and how they performed in the three different tuning techniques, please refer to Table 3. You can also find graphical representations of the algorithm performance in each tuning technique in Supplementary material 2.

**Table 3:** Accuracy and AUC value of the selected machine algorithms after data balancing and optimized with across different hyperparameter tuning techniques.

**Algorithms**	**Grid search**		**Random search**		**Baysian optimization**	
	**Accuracy**	**AUC**	**Accuracy**	**AUC**	**Accuracy**	**AUC**
SVM	91.0%	0.96	90.0%	0.96	95.0%	0.98
GNB	80.0%	0.88	80.0%	0.88	80.0%	0.88
Logistic regression	84.0%	0.91	84.0%	0.91	84.0%	0.92
Decision tree	87.0%	0.86	86.0%	0.88	83.0%	0.90
Random forest	92.0%	0.98	93.0%	0.98	83.0%	0.93
GBM	91.0%	0.97	90.0%	0.96	74.0%	0.84
XGBoost	90.0%	0.97	92.0%	0.97	70.0%	0.95
KNN	92.0%	0.96	93.0%	0.92	90.0%	0.96
MLP	89.0%	0.94	89.0%	0.94	90.0%	0.95
AdaBoost	85.0%	0.92	85.0%	0.92	85.0%	0.93
CatBoost	91.0%	0.97	92.0%	0.97	95.0%	0.98
ANN	89.0%	0.94	89.0%	0.94	89.0%	0.95

The bold and underlined numbers indicate the highest accuracy and AUC scores of each machine learning algorithm after comparison through various optimization techniques.

### Model interpretability and feature relevance

#### Association rule mining

By employing the Apriori algorithm, our study discovered seven influential association rules based on their lift values and confidence. Significantly, the consistent presence of variables such as maternal and paternal education levels, timing of the first ANC checkup during the first trimester (early ANC visit), wealth status, having marital status, mobile phone ownership, religious affiliation (Jehova or traditional), having media exposure, and giving birth for the first time indicated their strong association with the likelihood of facility delivery.

The top seven association rules and their corresponding lift values are as follows:

1. If mothers have a higher education level and their husbands also have a higher education level, the probability of giving birth at a facility is 97.4% (confidence = 0.974 and lift = 1.226).
2. If mothers have a higher education level and attend their first ANC checkup in the first trimester, the probability of giving birth at a facility is 95.8% (confidence = 0.958 and lift = 1.205).
3. If mothers are in the middle level of economic status and their husbands have a higher education level, the probability of giving birth at a facility is 95.5% (confidence = 0.955 and lift = 1.202).
4. If mothers and their husbands have a higher educational level and the mothers are married, the probability of giving birth at a facility is 96.7% (confidence = 0.967 and lift = 1.216).
5. If mothers have a higher educational level, own a mobile phone, and follow the Jehova religion, the probability of giving birth at a facility is 97.6% (confidence = 0.976 and lift = 1.228).
6. If mothers have a higher education level, have access to media, and follow the Jehova religion, the probability of giving birth at a facility is 97.5% (confidence = 0.975 and lift = 1.217).
7. If mothers have a higher education level, are giving birth for the first time, and follow a traditional religion, the probability of giving birth at a facility is 96.8% (confidence = 0.968 and lift = 1.219).

#### Evaluation of feature relevance

We used the CatBoost algorithm to analyze the importance of features in predicting the place of delivery. Choosing CatBoost over SVM, despite their equal prediction performance in this study, was due to CatBoost's interpretability advantage. Unlike SVM, CatBoost allows for direct interpretation of feature importance.

**Figure 7:**
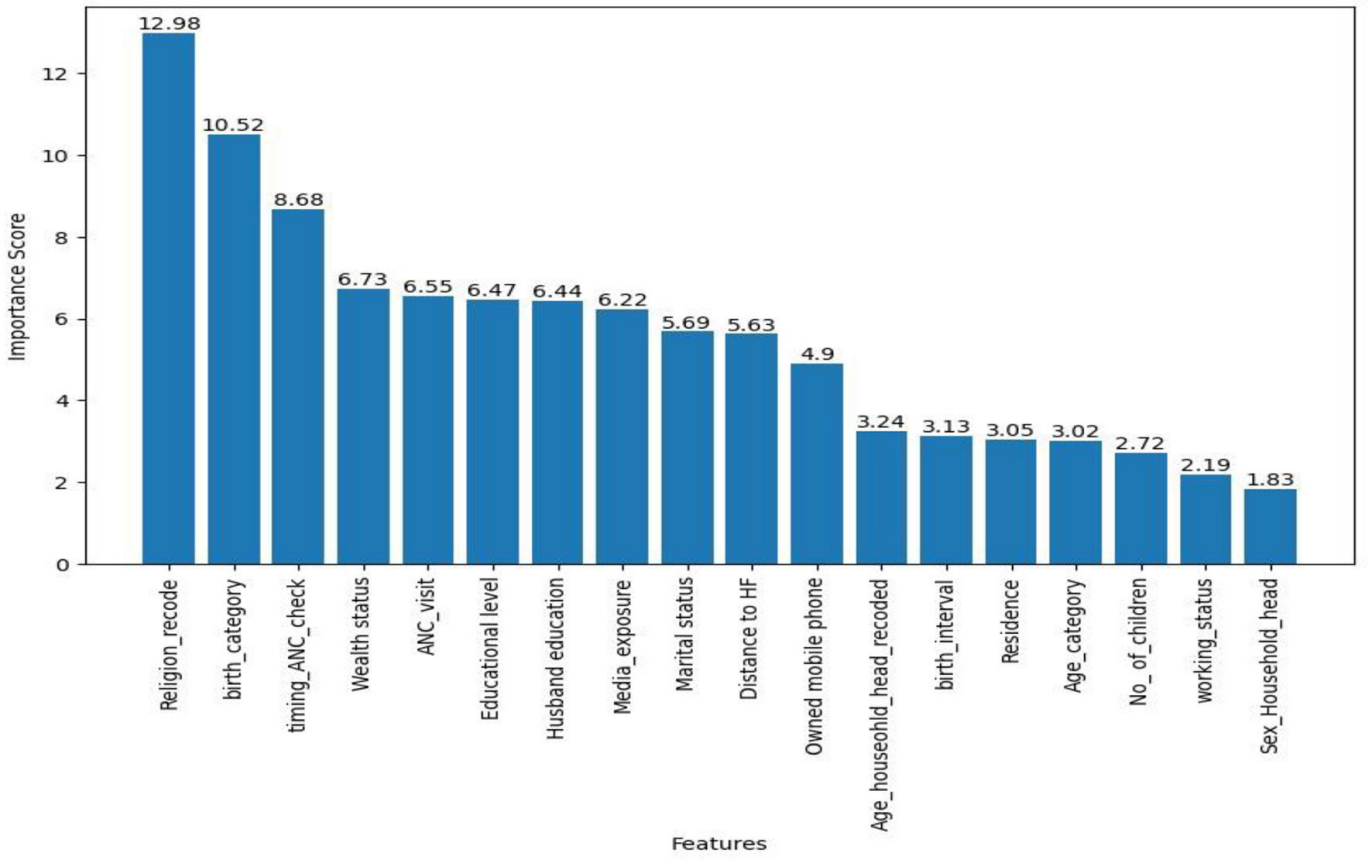
CatBoost based feature relevance.

Accordingly, the top seven importance variables for this prediction were religion, birth order category, timing of the first ANC checkup, wealth status, ANC visit, highest educational level of mother, and husband education level (see Figure 7).

## Discussion

In our research study conducted using recent DHS data of East African countries, we evaluated 12 advanced machine learning algorithms using different techniques to balance the data and fine-tune the hyperparameters. Our extensive experimentation enabled us to take the possible best performance metrics score of each algorithm after comparison in each data balancing and tuning technique.

Our analysis revealed that the overall prevalence of health facility delivery among women of reproductive age in East Africa was found to be 83.71% (95% CI: 83.48, 83.93). This figure is slightly lower than the previously reported rate of 87.49% (30). The discrepancy may stem from the different timeframes in which the surveys were conducted across countries. The decline in health facility deliveries signals the need for urgent measures to meet SDGs aimed at reducing maternal mortality.

Ethiopia exhibited the lowest health facility delivery rate at 38.05%, while Malawi had the highest at 94.35%. The challenges in Ethiopia may relate to insufficient medical care and human resources to serve over 120 million people (93).

Out of the 12 algorithms that were evaluated and compared, SVM and CatBoost after being optimized using Bayesian optimization tuning emerged as the top performers in predicting the place of delivery, in which both of those algorithms scored an accuracy of 95%, and AUC of 0.98. Furthermore, a comprehensive analysis of various performance metrics indicated that there was no significant difference between these two algorithms, highlighting their comparable capabilities in predicting the place of delivery. It is worth noting that the performance of these algorithms in our study surpasses that of previous research on predicting the place of delivery (59–61). This variation could be attributed to the unique nature of our dataset, which involved a larger sample size and different features. Additionally, our study focused on extensively experimenting with various data balancing and tuning techniques, which may have contributed to the improved performance as noted by previous scholarly findings in this field (76, 77, 85, 86).

On the other hand, the performance of the top-performing algorithms in our study was lower compared to previous research in Ethiopia (62) that focused on predicting skilled delivery. This disparity could be attributed to differences in dataset characteristics and the methodology employed for feature selection. While the previous Ethiopian study solely utilized variables identified as significant in the multivariable logistic regression for developing their model, we employed the Boruta algorithm feature selection technique after comparing various techniques. This difference in feature selection methodology could potentially explain the variation in the performance of the machine learning algorithms.

Our findings implied the effectiveness of SVM and CatBoost when optimized with Bayesian optimization tuning. SVM combines the strengths of effective classification with the efficiency of Bayesian optimization for hyperparameter optimization, resulting in improved predictions. Similarly, CatBoost, designed for categorical data, performs well when paired with Bayesian optimization tuning due to its ability to handle categorical features and benefit from optimization capabilities. This finding is supported by several studies conducted by different researchers who analyze the strengths and weaknesses of each algorithm (94–96).

Association rule mining analysis led us to identify seven strong rules with confidence above 95.0% that provide insights into the factors influencing facility-based deliveries. One consistent finding is that higher education levels of both mothers and their husbands are strongly associated with an increased probability of delivering at a facility. This association can be explained by the fact that education equips individuals with knowledge about the benefits of skilled birth attendance and the risks associated with home deliveries. Educated individuals are more likely to understand the importance of accessing healthcare facilities for safe deliveries and are empowered to make informed decisions. The finding is supported by previous studies (33).

The findings indicate that early ANC checkups are a significant predictor consistently identified in the association rules. This suggests that early ANC visits influence women's decisions regarding facility delivery. During these visits, expectant mothers receive essential information about the importance of skilled birth attendance and are encouraged to deliver in healthcare facilities. Notably, this finding aligns with a study conducted in Southern Ethiopia (37), though it contradicts results from a study based on the Mini Ethiopian DHS (36).

We also find that wealth status plays a role in determining the probability of delivering at a health facility. The middle level of economic status is associated with facility delivery, highlighting the role of financial resources in accessing and utilizing maternal healthcare services. This could be due to women with good economic status being more likely to afford the costs associated with facility deliveries, including transportation, medical fees, and other related expenses. Additionally, they may have access to better healthcare facilities, which further encourages facility-based deliveries. This finding is supported by previous studies elsewhere in the world (34).

Being married is found to enhance the chances of opting for facility delivery. This could be because married women often have a partner who can assist them in decision-making regarding childbirth, which may influence their choice to select facility delivery. This finding is supported by previous studies conducted in Northern Ethiopia, which highlight the potential link between marital status and the utilization of maternal health services (97).

Mobile phone ownership is significantly associated with facility delivery. This correlation likely stems from mobile phones' ability to provide access to health-related information, helping women gather crucial knowledge about the benefits of facility delivery and the services available. While ownership does not necessarily imply internet access or that one has a smartphone only, it still enhances communication with healthcare providers through features such as voice calls and text messaging. This functionality enables women to seek advice and assistance during their decision-making process. This finding aligns with a previous study conducted elsewhere (42).

Religious affiliation, specifically being a follower of Jehovah or traditional religions, is also associated with a higher likelihood of facility delivery. This finding suggests the importance of considering religious factors in public health interventions and highlights the need for targeted, culturally sensitive strategies to enhance health facility delivery in diverse religious communities. The finding is supported by studies elsewhere in the world (31).

Media exposure is consistently linked to a higher probability of delivering at a health facility. This could be due to media platforms, such as radio, television, or the internet, playing a crucial role in disseminating information about the benefits of facility delivery, available services, and success stories. Exposure to such messages creates awareness and helps women make informed choices regarding their place of delivery. This finding is supported by previous studies elsewhere in the world (29, 31).

According to the top seven association rules identified in this study, give a first-order child increase the likelihood of facility-based deliveries. This finding is supported by studies conducted elsewhere (42, 43). A possible explanation is that first-time mothers may be more aware of the potential complications associated with home deliveries, leading them to choose facility-based care for their childbirth.

### Strengths and limitations of the study

This study has several notable strengths. It conducted a comprehensive evaluation of 12 advanced machine learning algorithms using a relatively large dataset, thoroughly examining their performance. Additionally, extensive experimentation with various data balancing and hyperparameter tuning techniques was undertaken to optimize each algorithm's effectiveness, enhancing the reliability of the findings. The study also identified key factors associated with facility-based deliveries, providing valuable insights for interventions and strategies.

However, certain limitations must be considered when interpreting the results. Firstly, the study relied on existing survey data, which may have inherent limitations and gaps in capturing some relevant variables. Another limitation is the lack of exploration of ensemble techniques, which combine multiple models to improve predictive accuracy. The study utilized DHS data starting from 2012, which may affect its applicability and highlights the opportunity for further validation with more recent data. Lastly, the exclusive use of the Apriori algorithm for constructing association rules presents a limitation.

## Conclusion

The analysis of various machine learning algorithms, alongside various techniques, revealed that the SVM and CatBoost algorithms excelled in accurately identifying health facility-based deliveries. These results underscore the effectiveness of machine learning models as valuable tools for healthcare providers and policymakers, enabling them to identify women at risk of delivering outside healthcare facilities and design targeted interventions to promote safe deliveries.

The overall prevalence of facility-based deliveries stands at 83.71%, indicating a slight decline from previous reports. This trend highlights the urgent need for targeted interventions to meet SDGs, particularly in maternal health.

Furthermore, the investigation into factors influencing facility-based deliveries through association rule mining identified several key determinants, including education level, early ANC visits, wealth status, marital status, mobile phone ownership, religious affiliation, media exposure, and giving birth for the first time. By addressing these factors through tailored interventions and policies, stakeholders can enhance health outcomes for mothers and children in East Africa.

This study recommends promoting facility-based deliveries through a variety of strategies: raising awareness about skilled birth attendance, encouraging early ANC visits, addressing financial barriers with targeted support programs, implementing culturally sensitive interventions, and utilizing media campaigns and mobile health initiatives. Additionally, interventions should be designed with consideration for the birth order of the child, recognizing that mothers may have different informational needs depending on whether it is their first or subsequent delivery.

Future research should consider additional contextual factors to develop a comprehensive understanding of influences on the place of delivery. Researchers are also encouraged to explore alternative algorithms for constructing association rules and to experiment with model ensembling techniques to optimize algorithm performance. Incorporating more recent and diverse datasets will further enhance the relevance and applicability of findings. By adopting these approaches, researchers can improve the accuracy and robustness of their predictions.

## Data Availability

Publicly available datasets were analyzed in this study. This data can be found at: the data used for this study can be accessed from the DHS website upon request to the appropriate authority (URL: https://dhsprogram.com/data/available-datasets.cfm). Additionally, the source code used for this study can be shared upon reasonable request from the correspondence author.
