# Supplementary material for: Predicting place of delivery choice among childbearing women in East Africa: a comparative analysis of advanced machine learning techniques
Source: Front Public Health. 2024 Nov 27;12:1439320. doi: 10.3389/fpubh.2024.1439320 (PMC11631870; doi:10.3389/fpubh.2024.1439320)

Performance metrics of each machine learning algorithm using unbalanced data

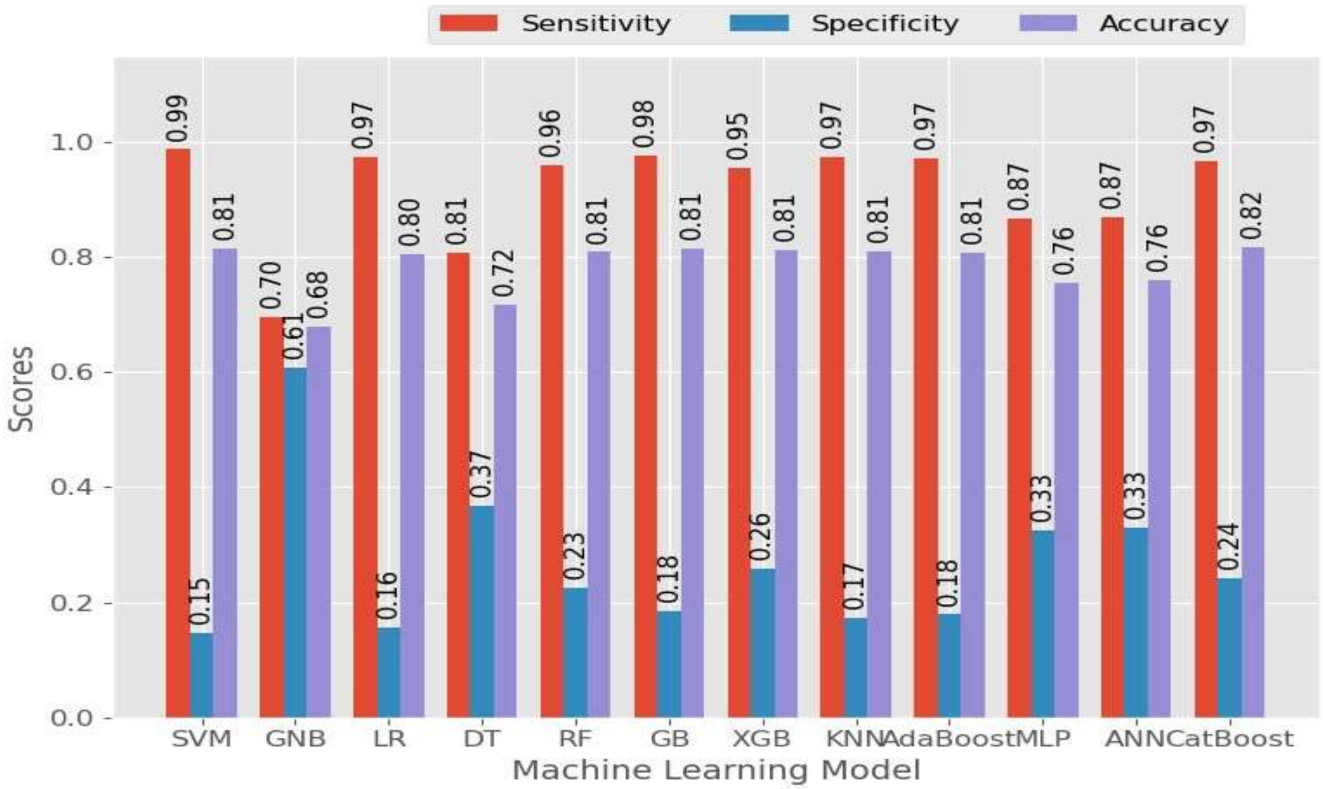

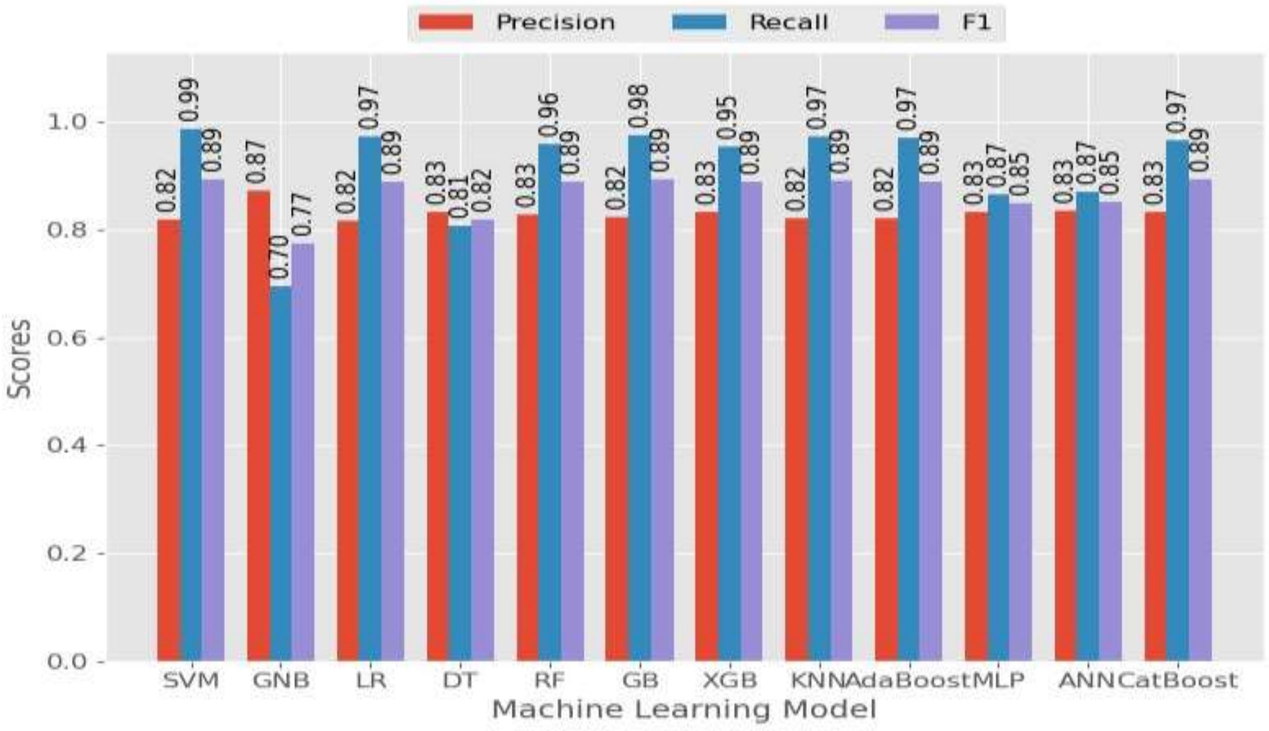

ROC curve value of each machine learning algorithm using unbalanced data

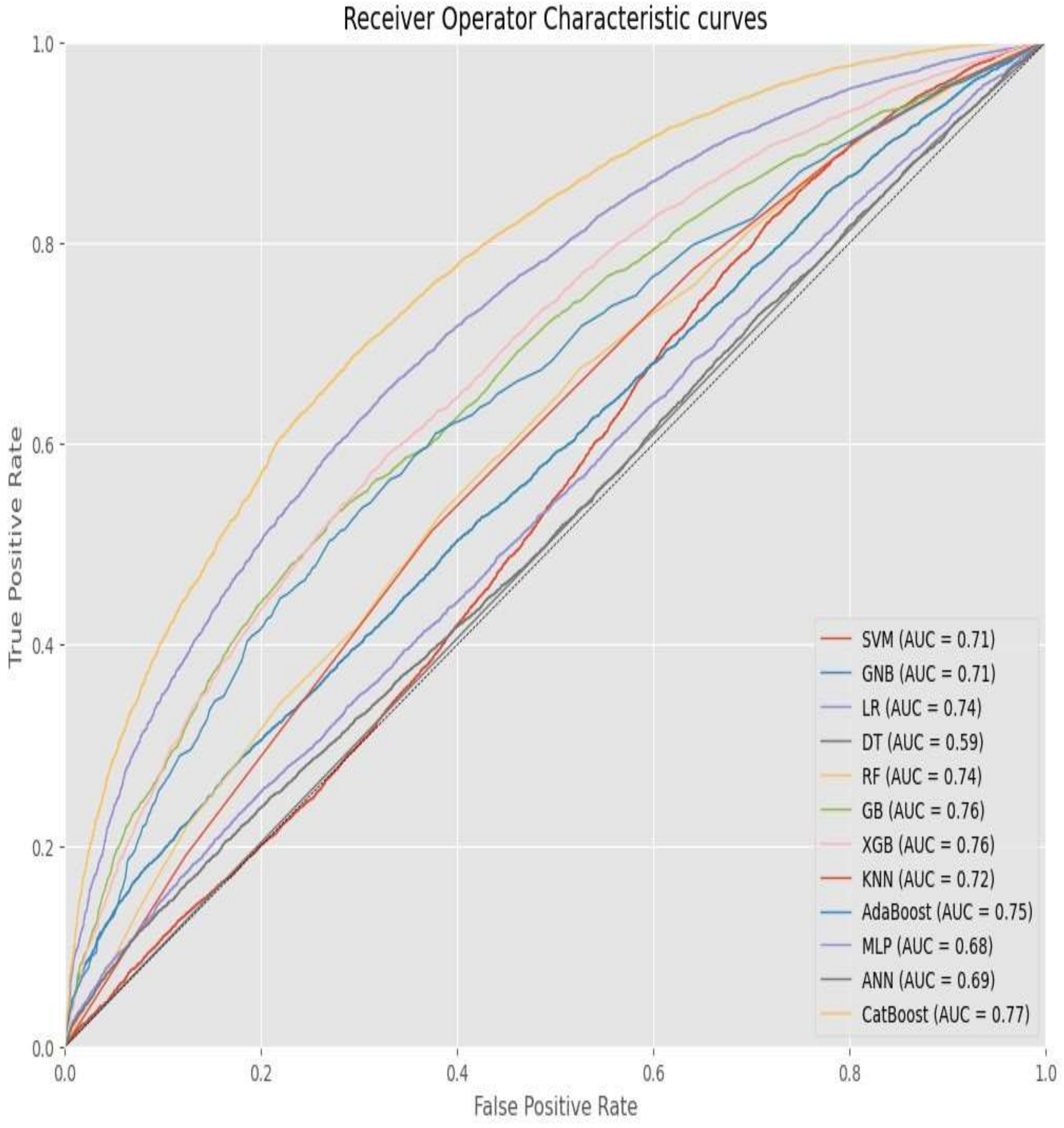

Performance metrics of each machine learning algorithm after data were balanced with under-sampling data balancing technique

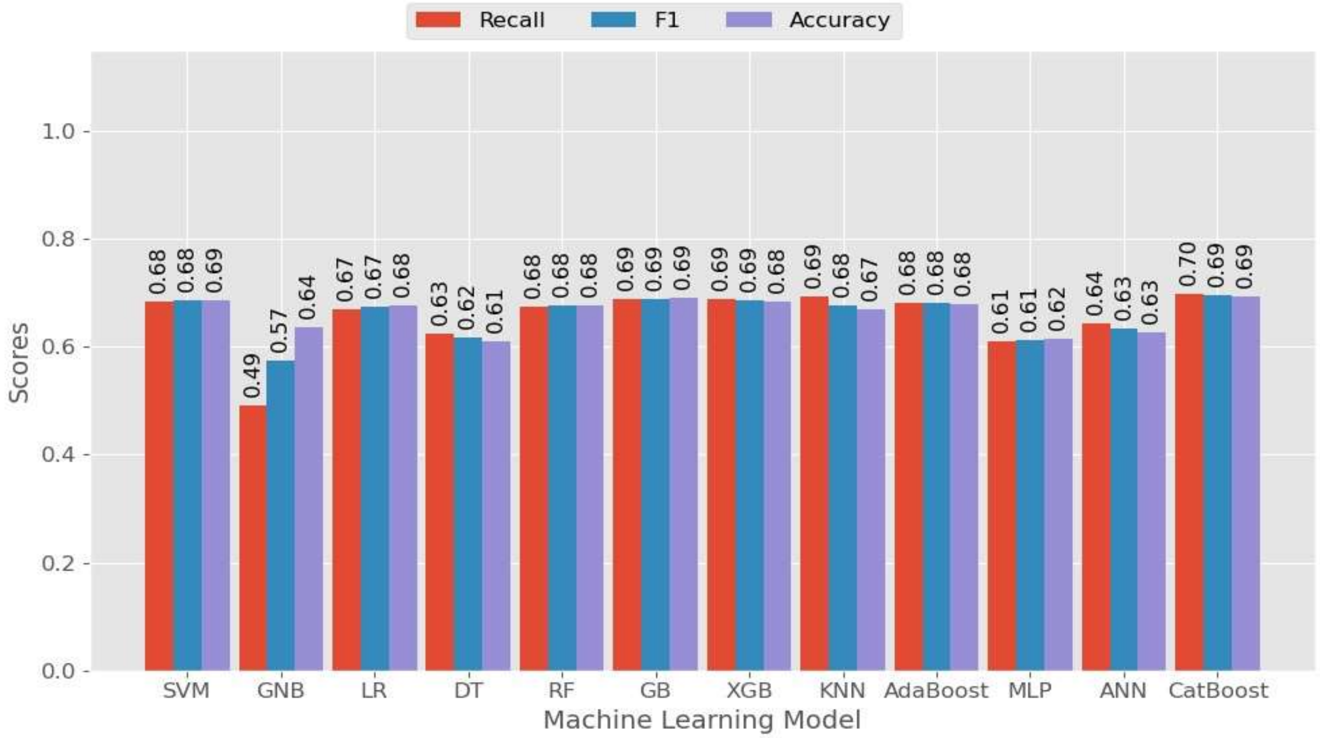

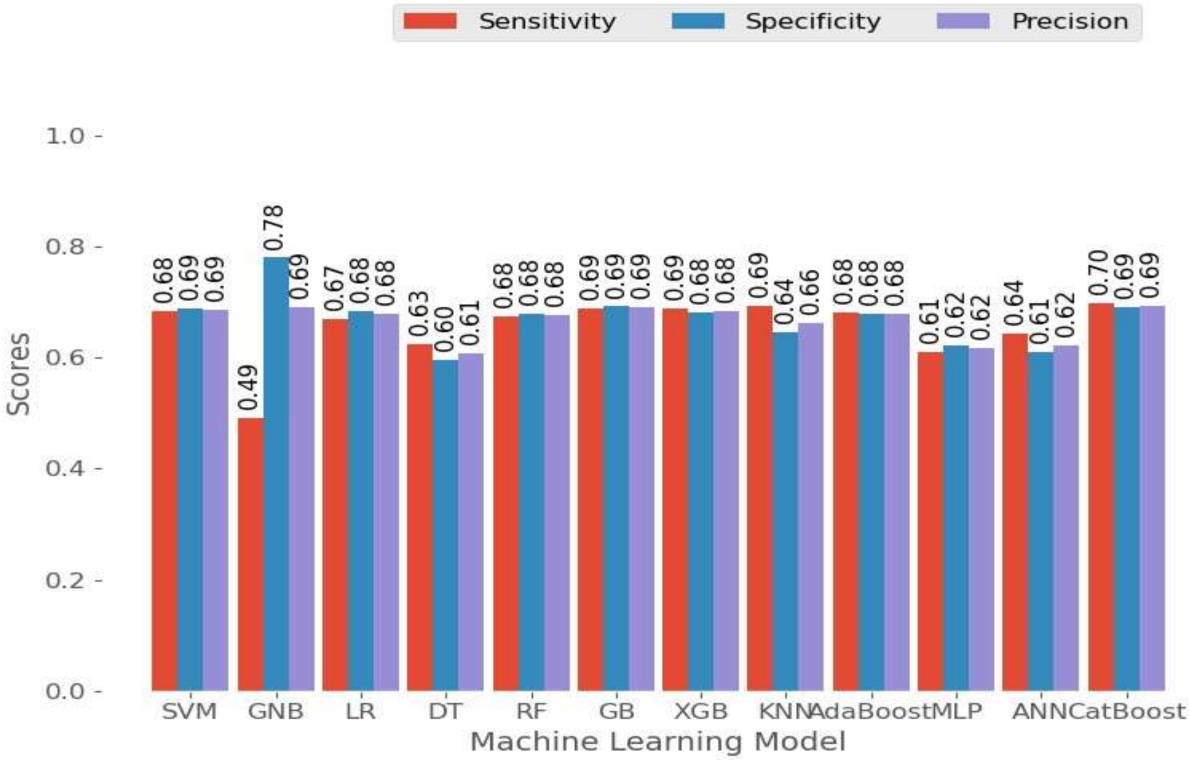

ROC curve value of each machine learning algorithm after data were balanced with under-sampling data balancing technique

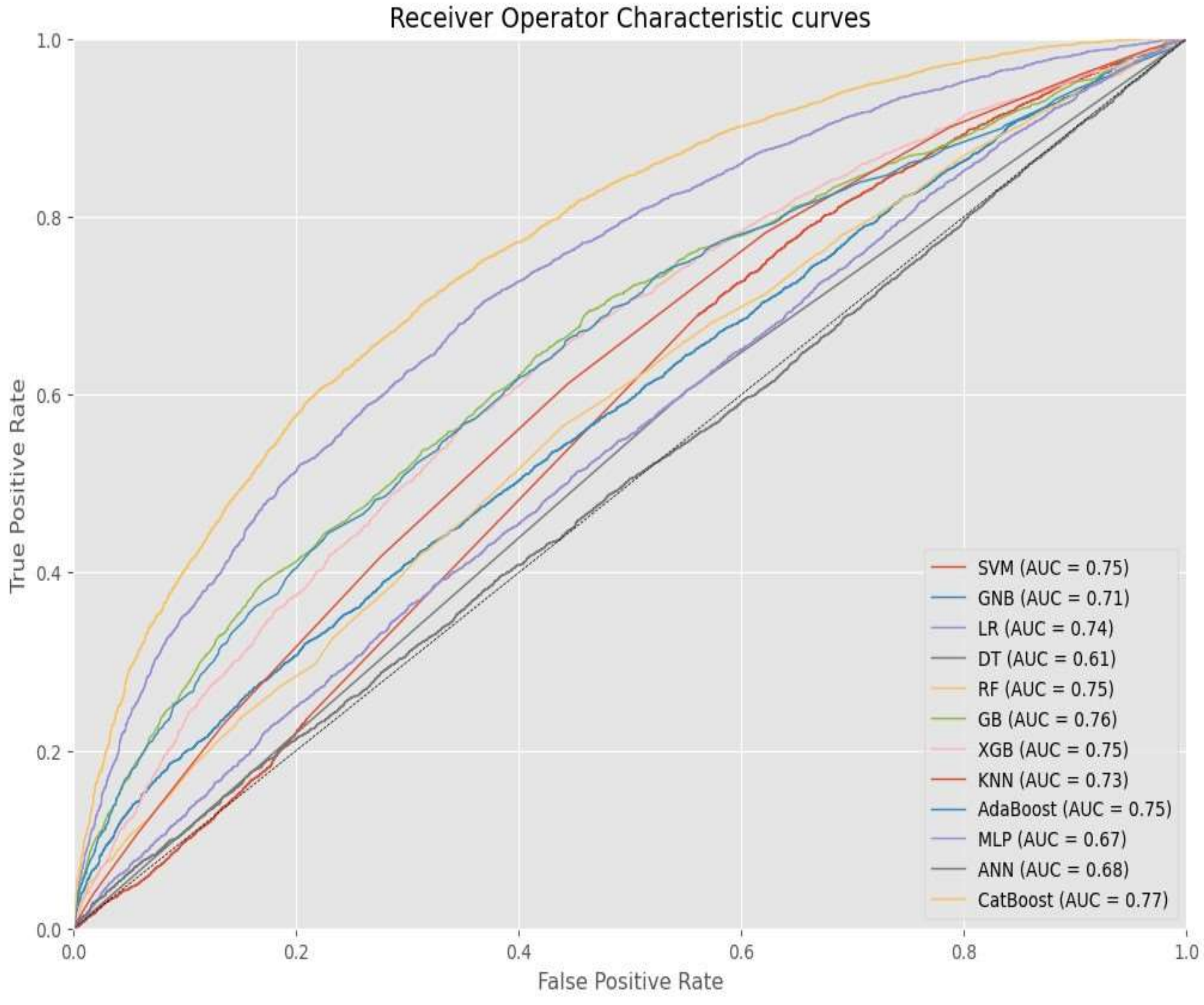

Performance metrics of each machine learning algorithm after data were balanced with over-sampling data balancing technique

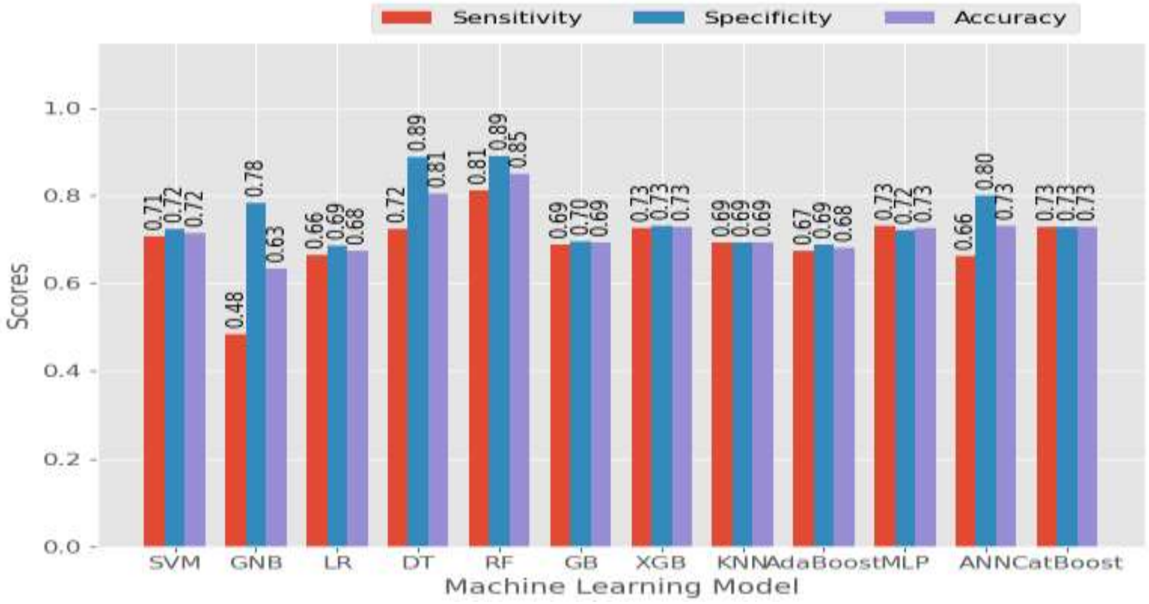

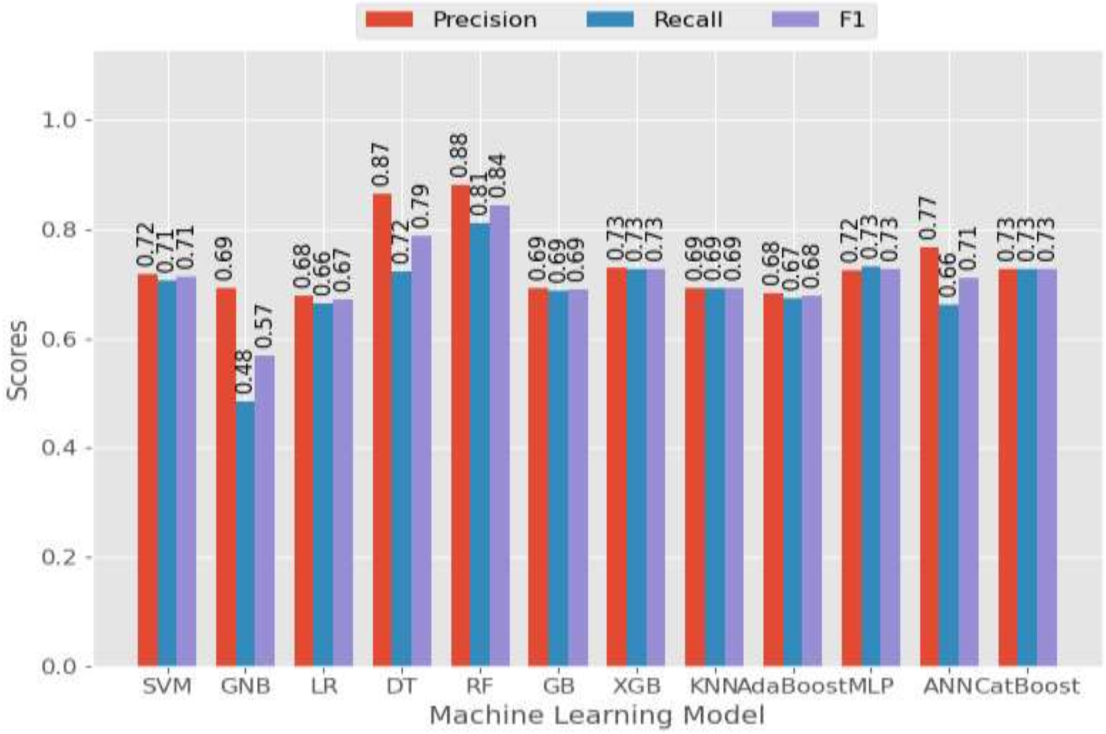

ROC curve analysis of each machine learning algorithm after data were balanced with over-sampling data balancing technique

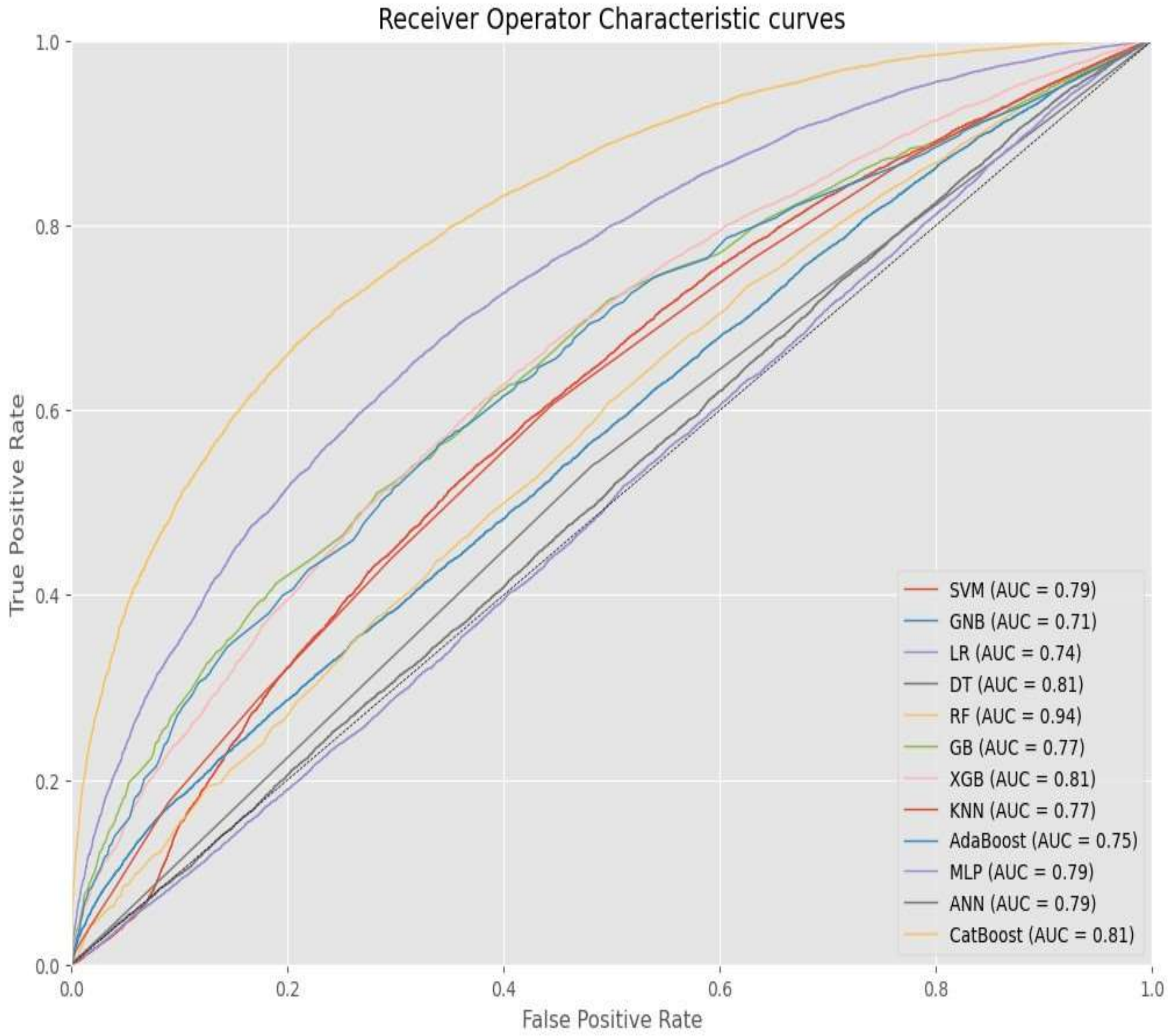

Performance metrics of each machine learning algorithm after data were balanced with SMOTE data balancing technique

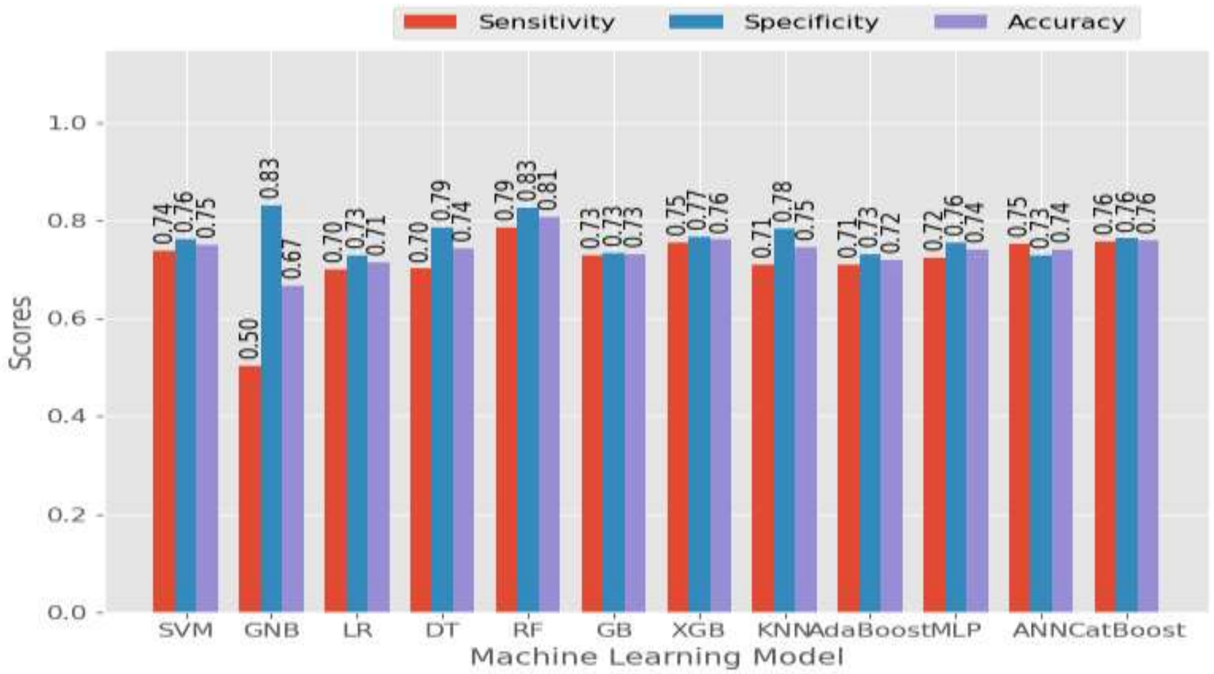

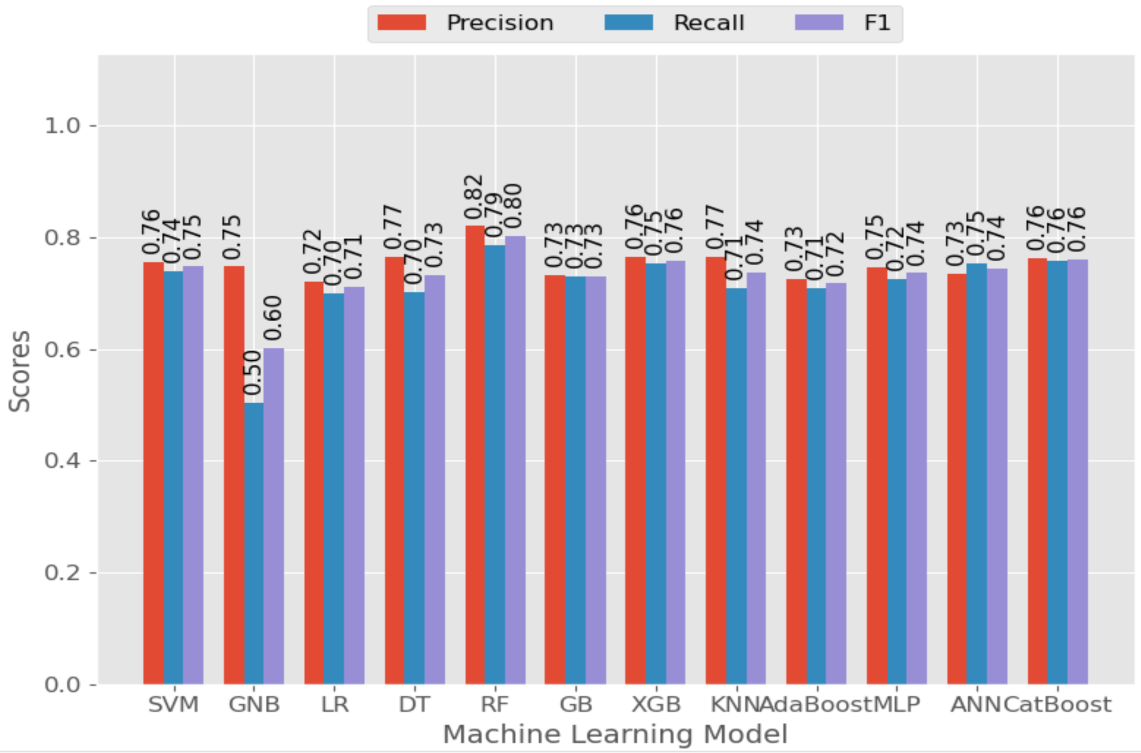

ROC curve value of each machine learning algorithm after data were balanced with SMOTE data balancing technique

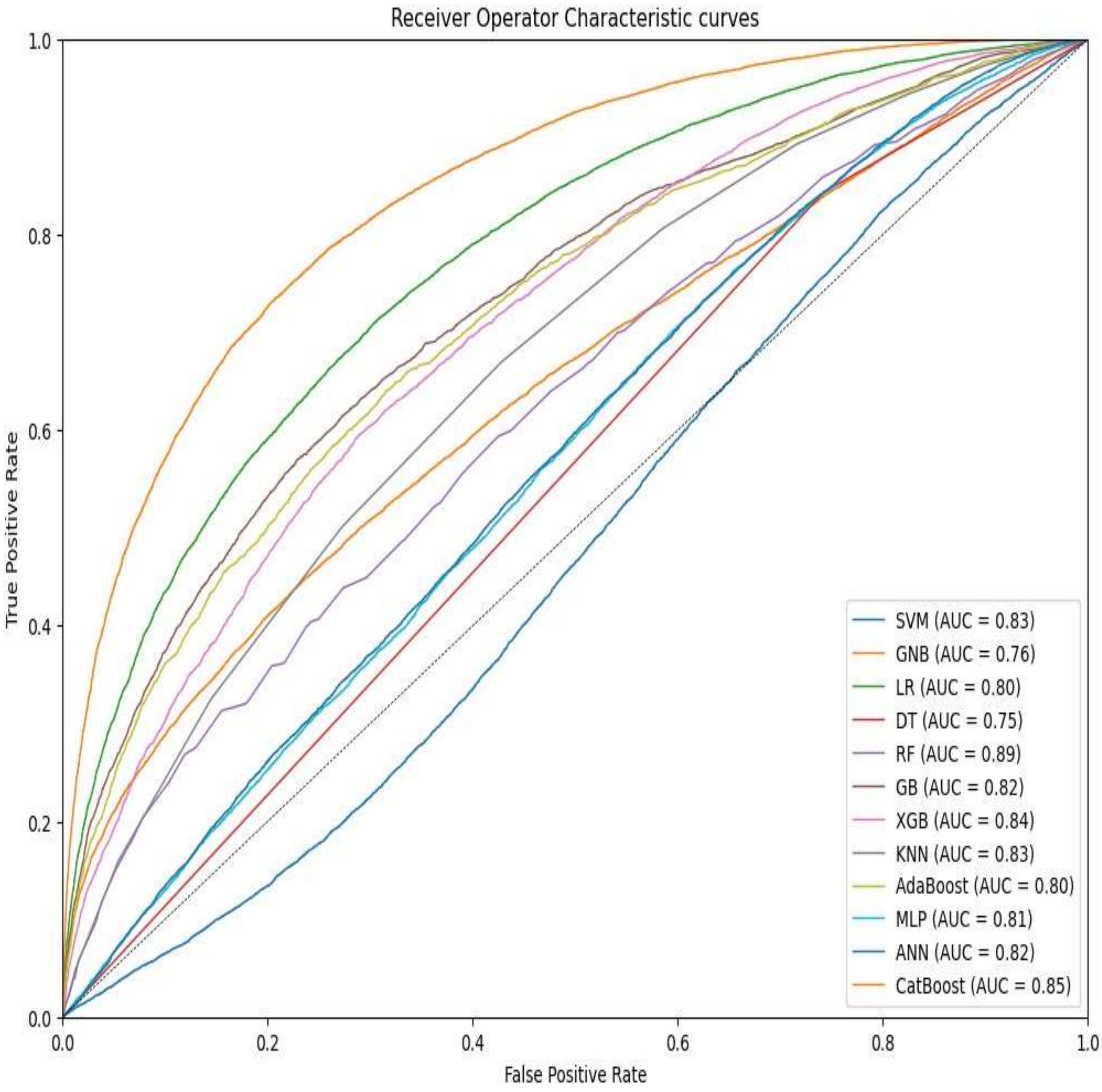

Performance metrics of each machine learning algorithm after data were balanced with ADASYN data balancing technique

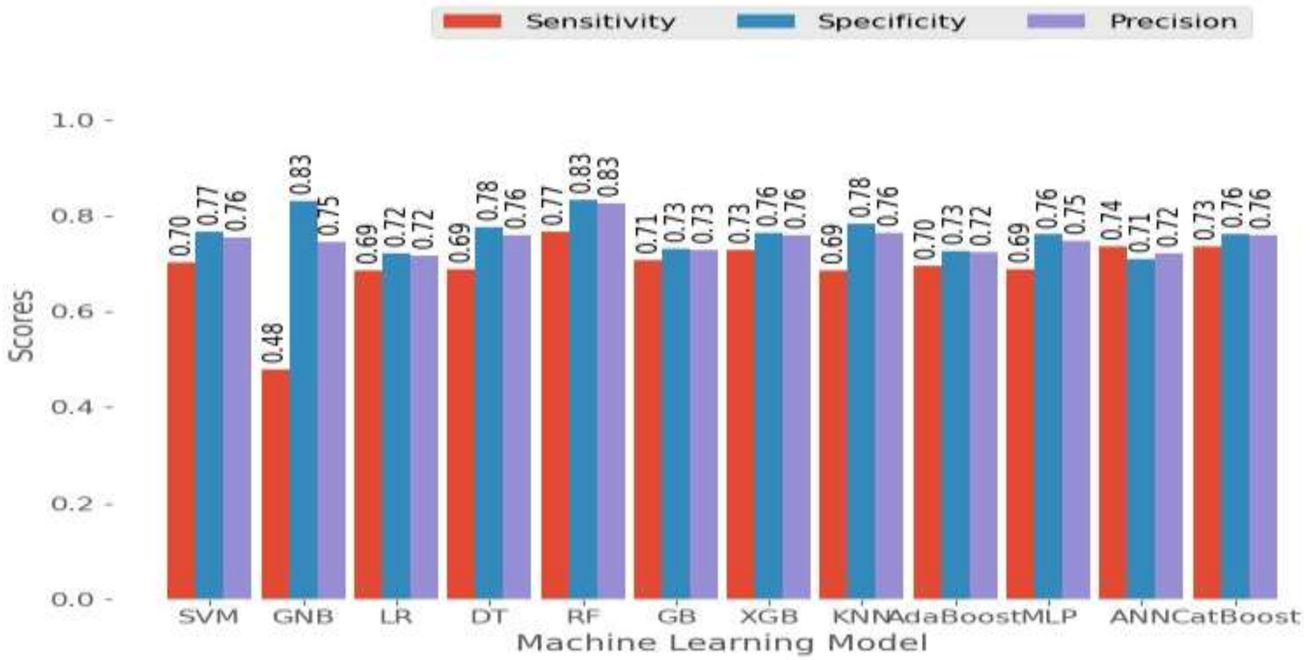

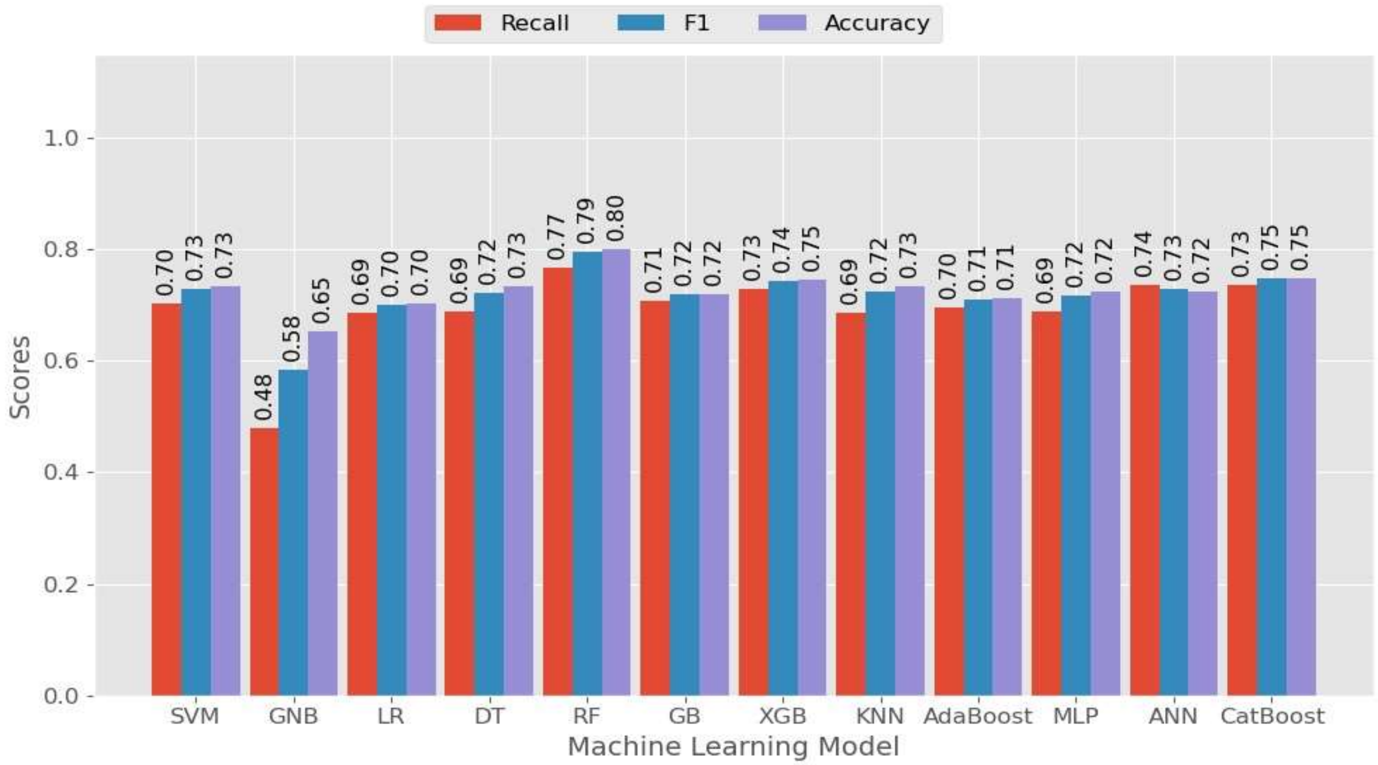

ROC curve value of each machine learning algorithm after data were balanced with ADASYN data balancing technique

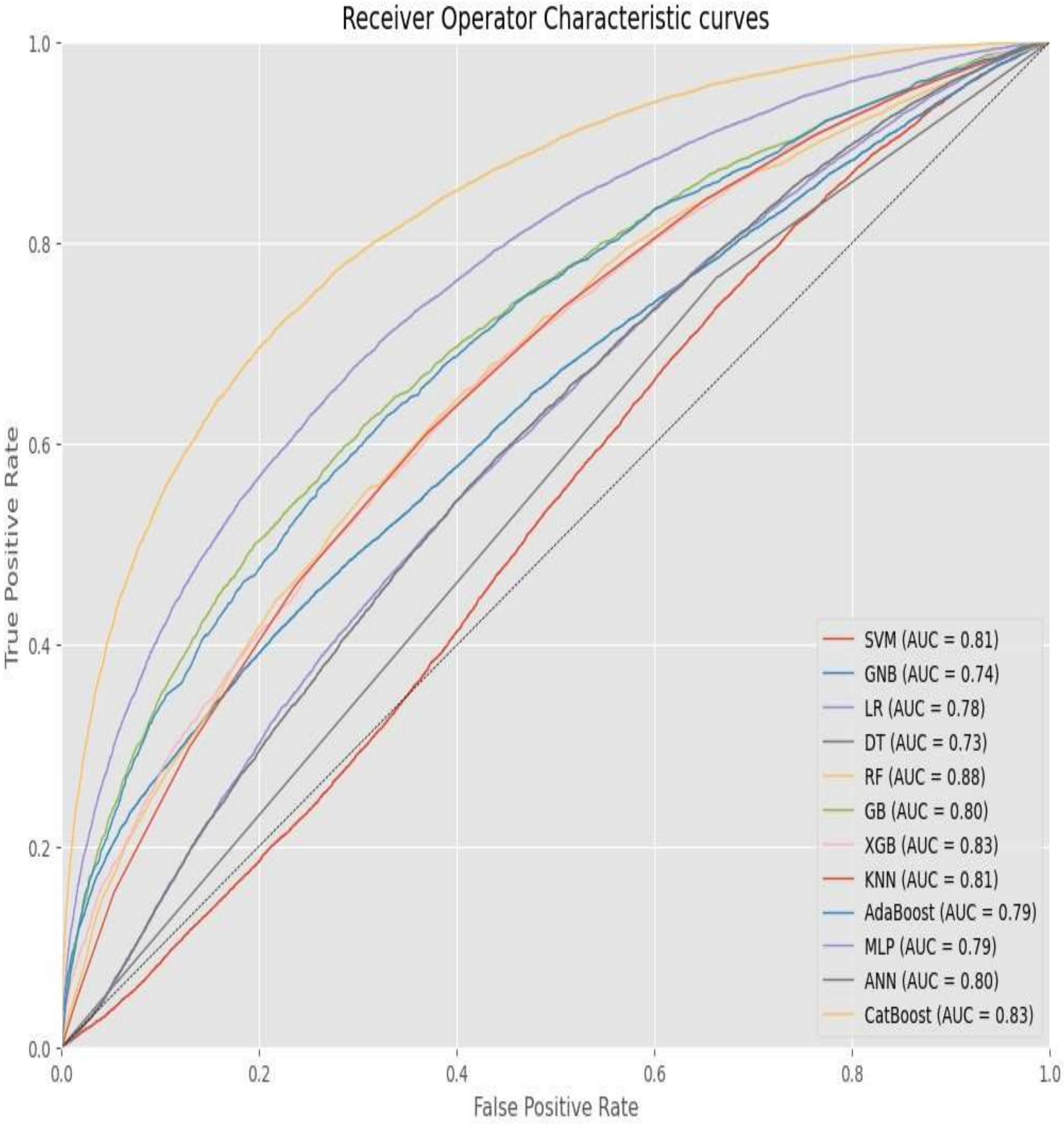

Performance metrics of each machine learning algorithm after data were balanced with SMOTE-ENN data balancing technique

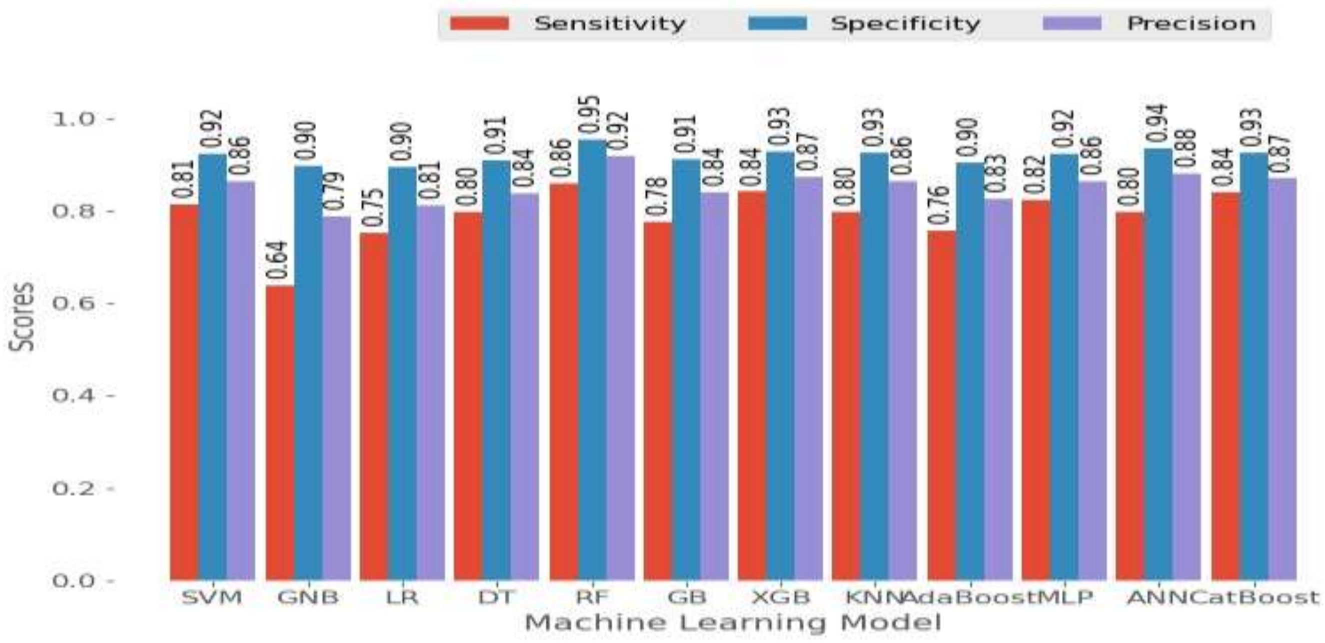

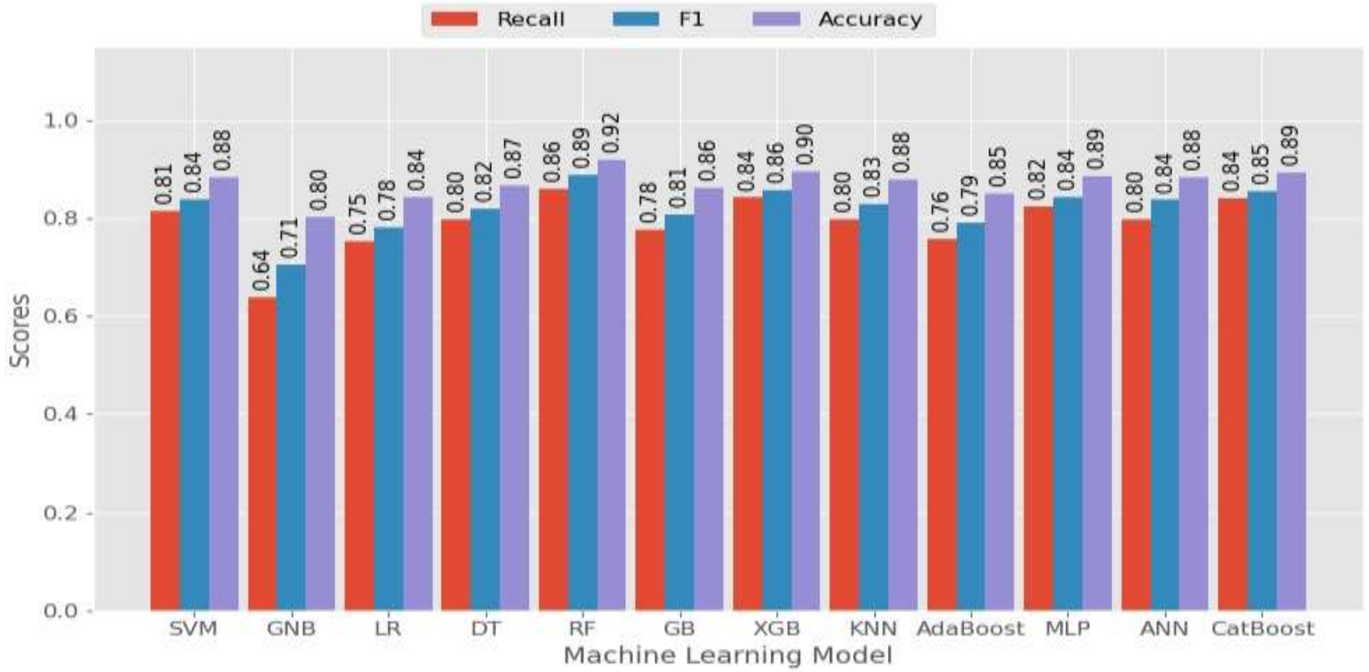

ROC curve value of each machine learning algorithm after data were balanced with SMOTE-ENN data balancing technique

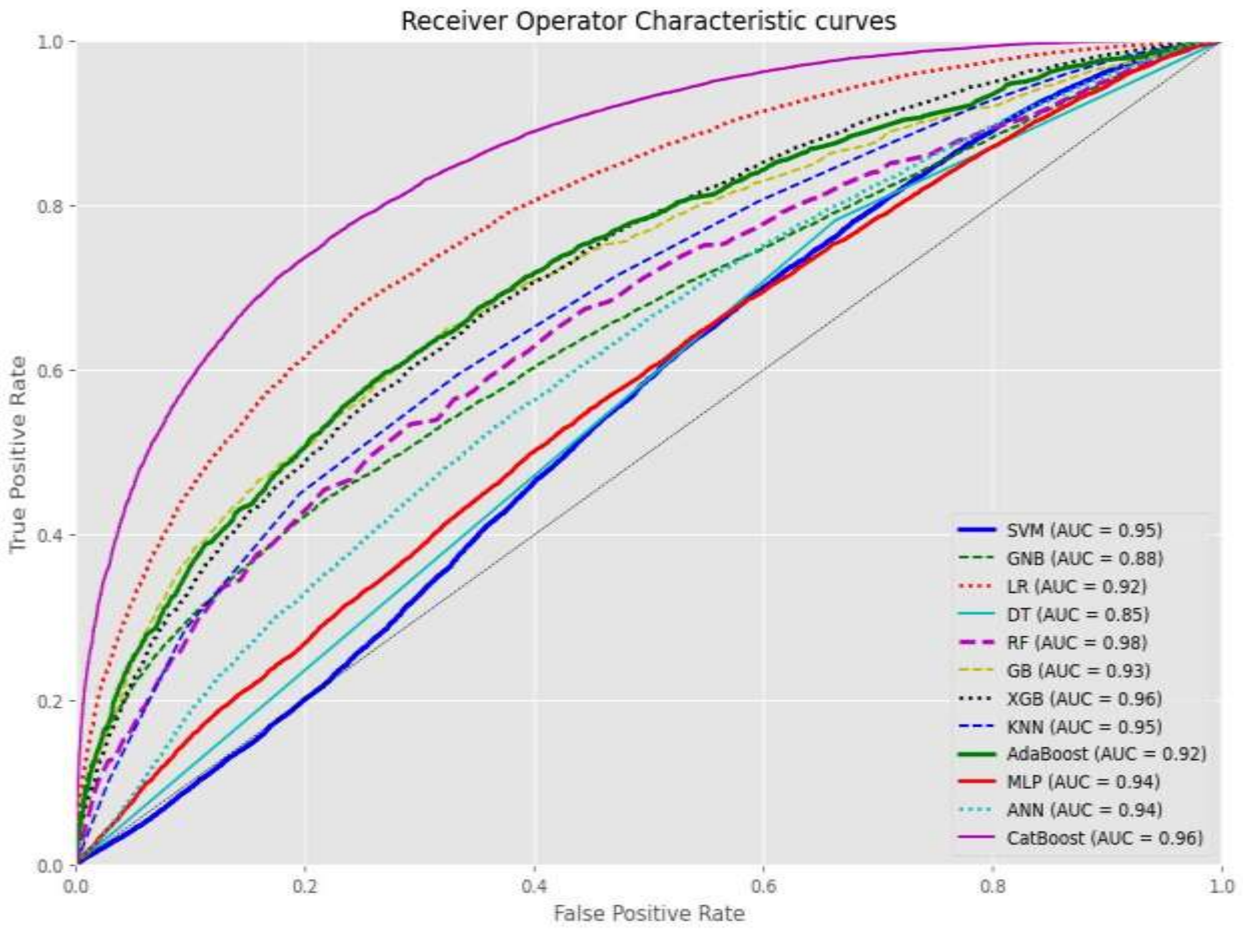

Performance metrics of each machine learning algorithm after data were balanced with Near-miss algorithm data balancing technique

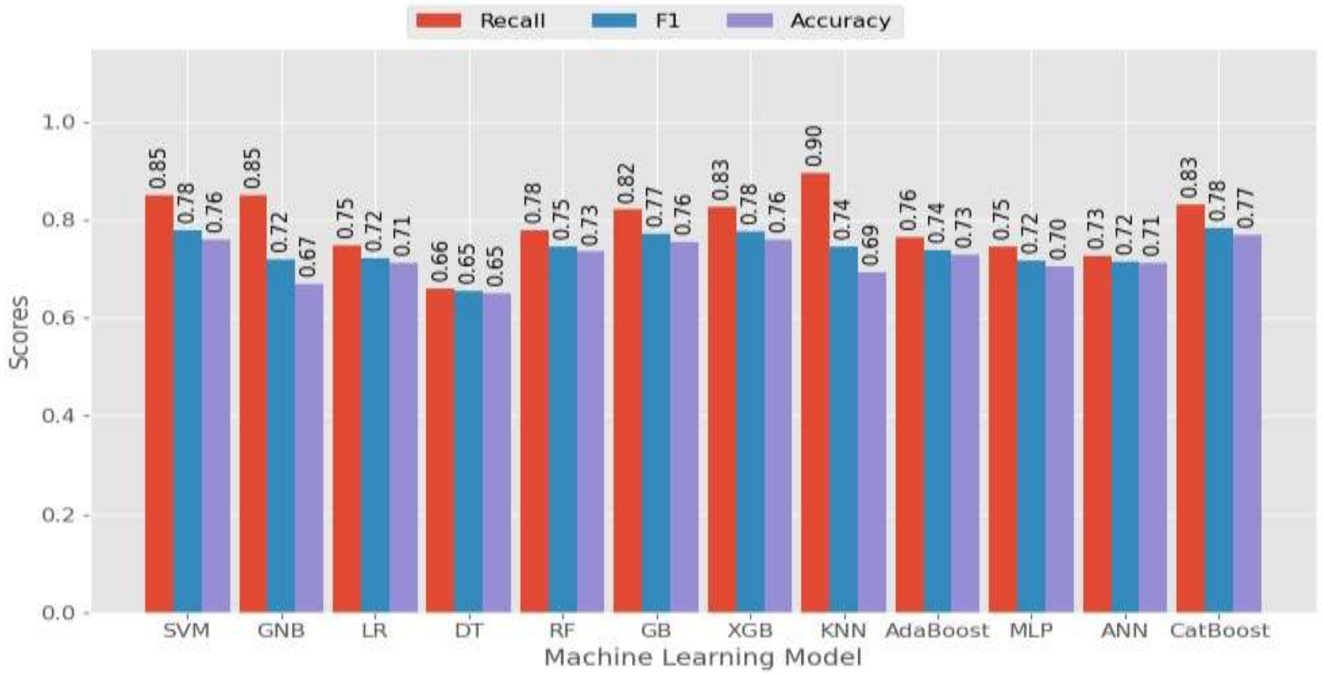

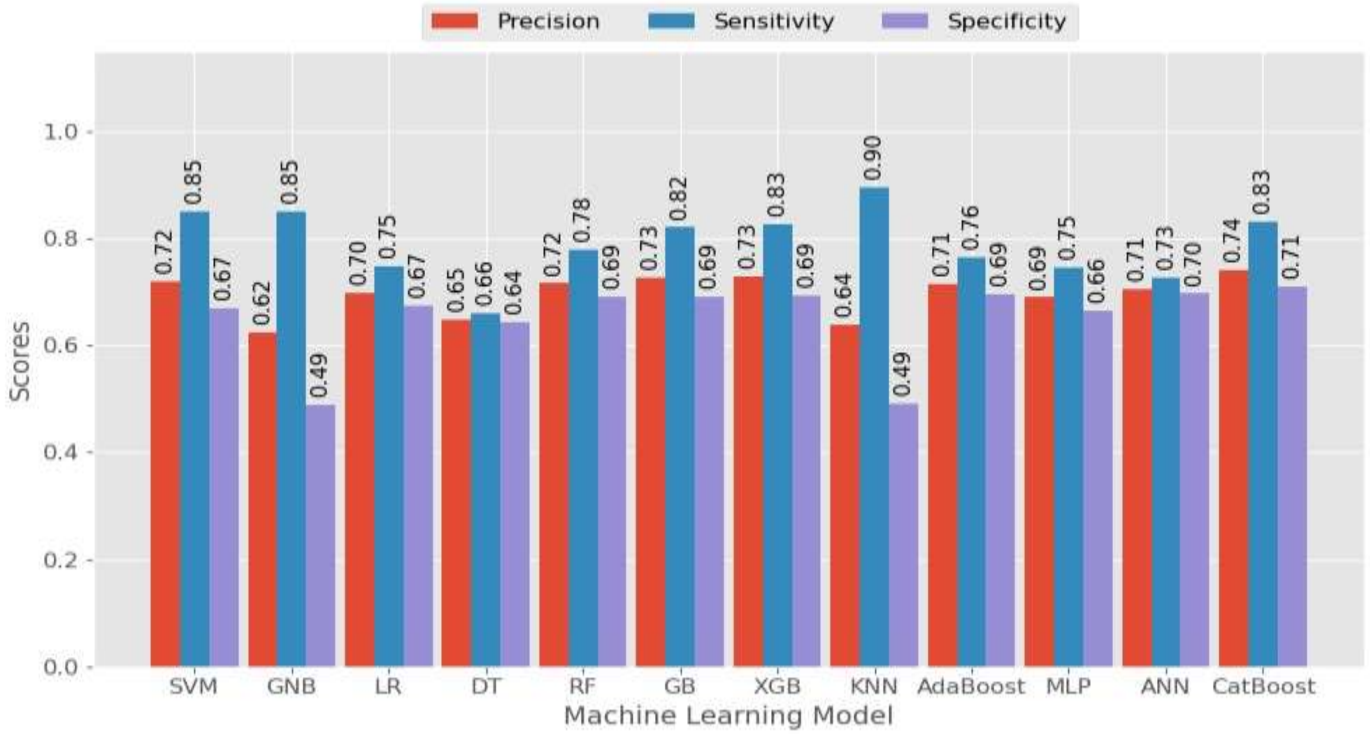

ROC curve value of each machine learning algorithm after data were balanced with Near-miss algorithm data balancing technique

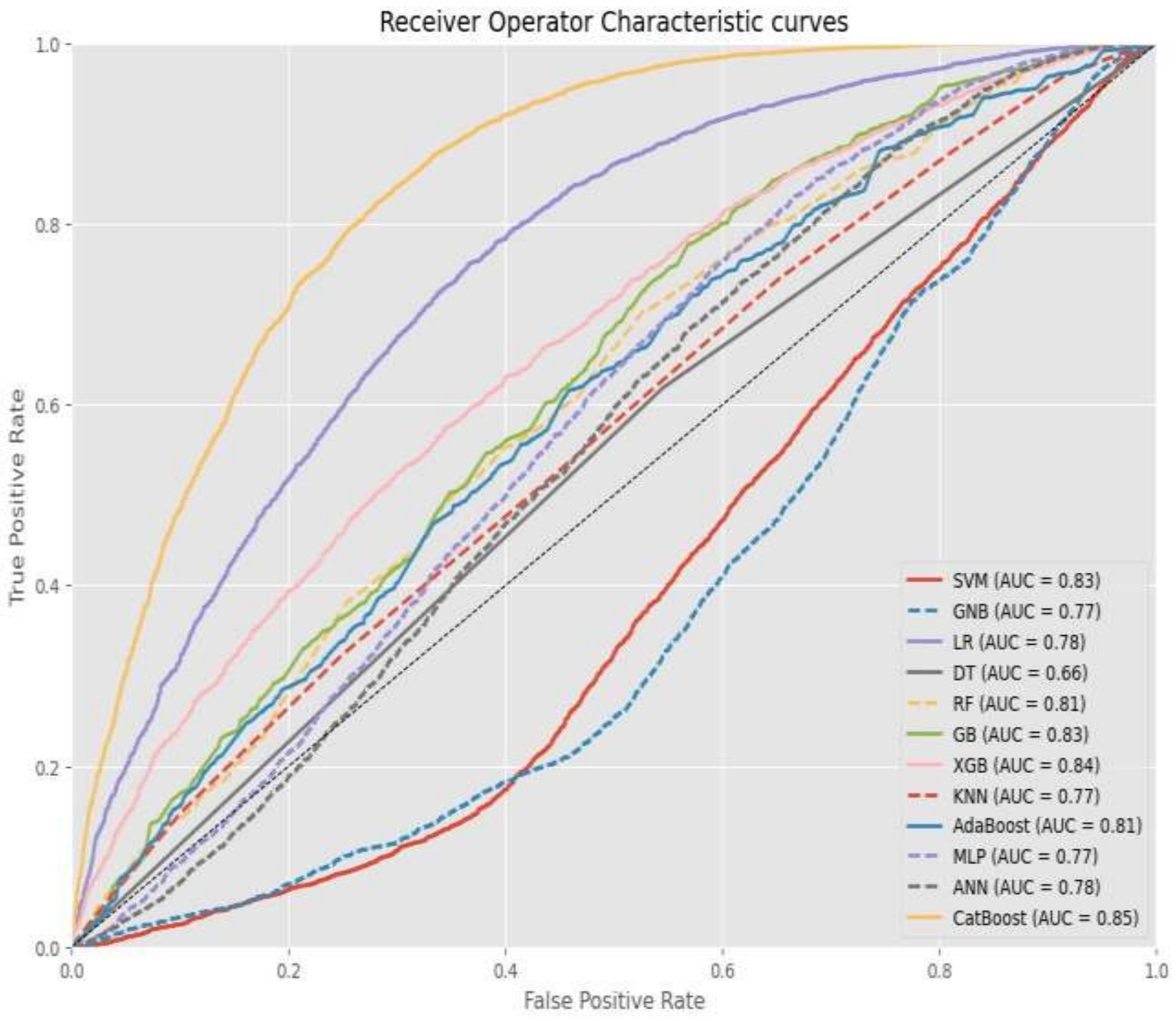

Performance metrics of each machine learning algorithm after data were balanced with SMOTE-Tomek data balancing technique

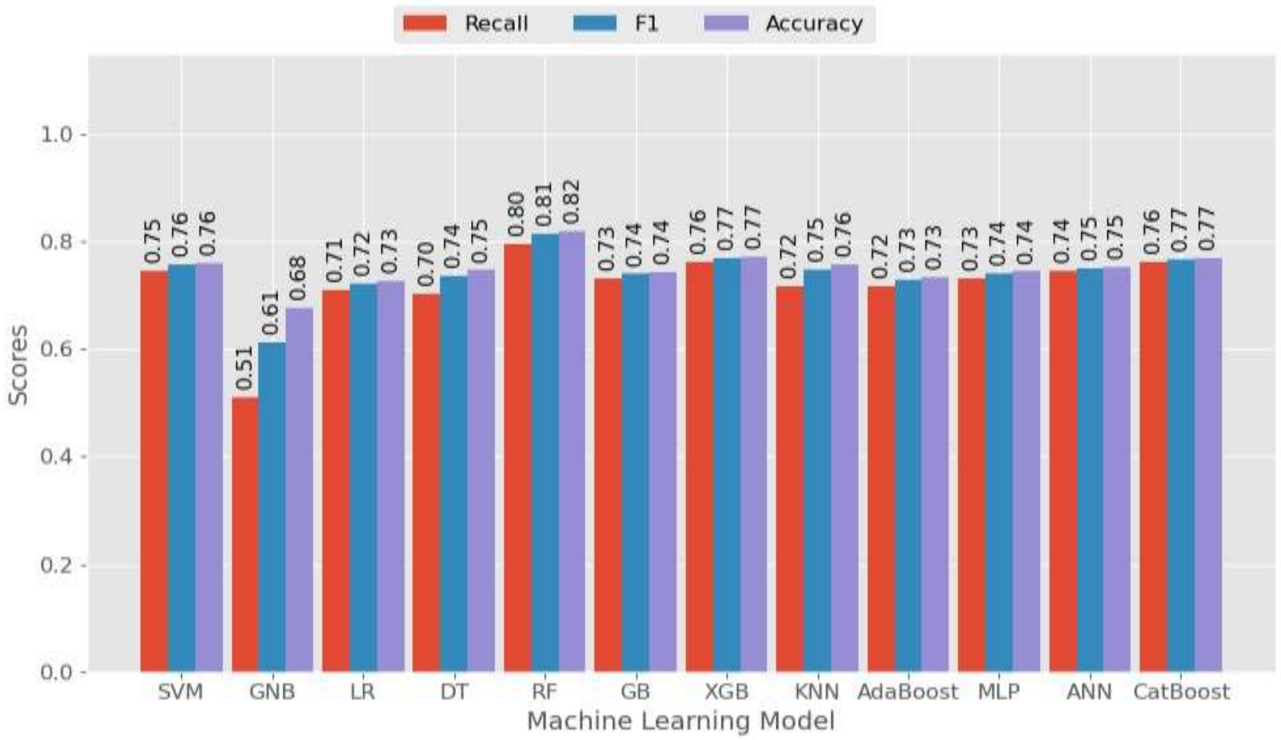

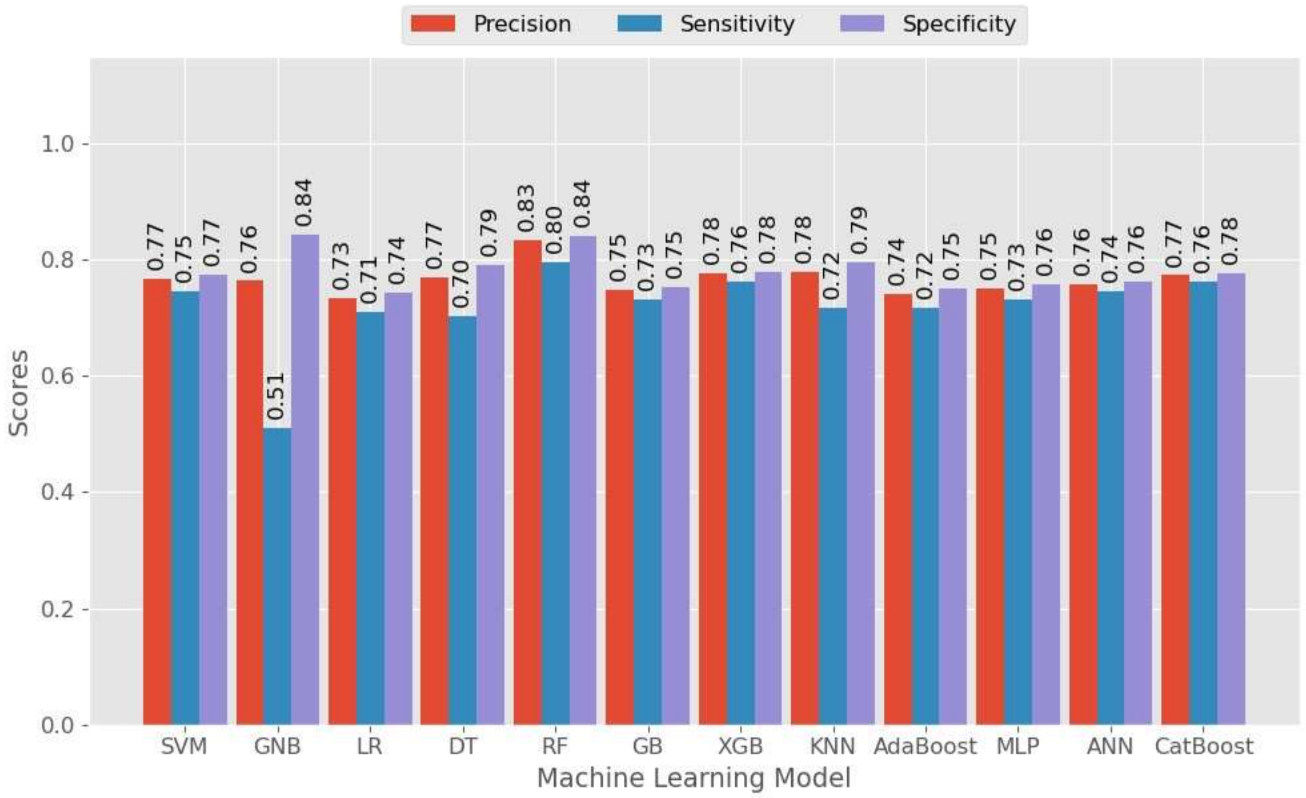

ROC curve value of each machine learning algorithm after data were balanced with SMOTE-Tomek data balancing technique

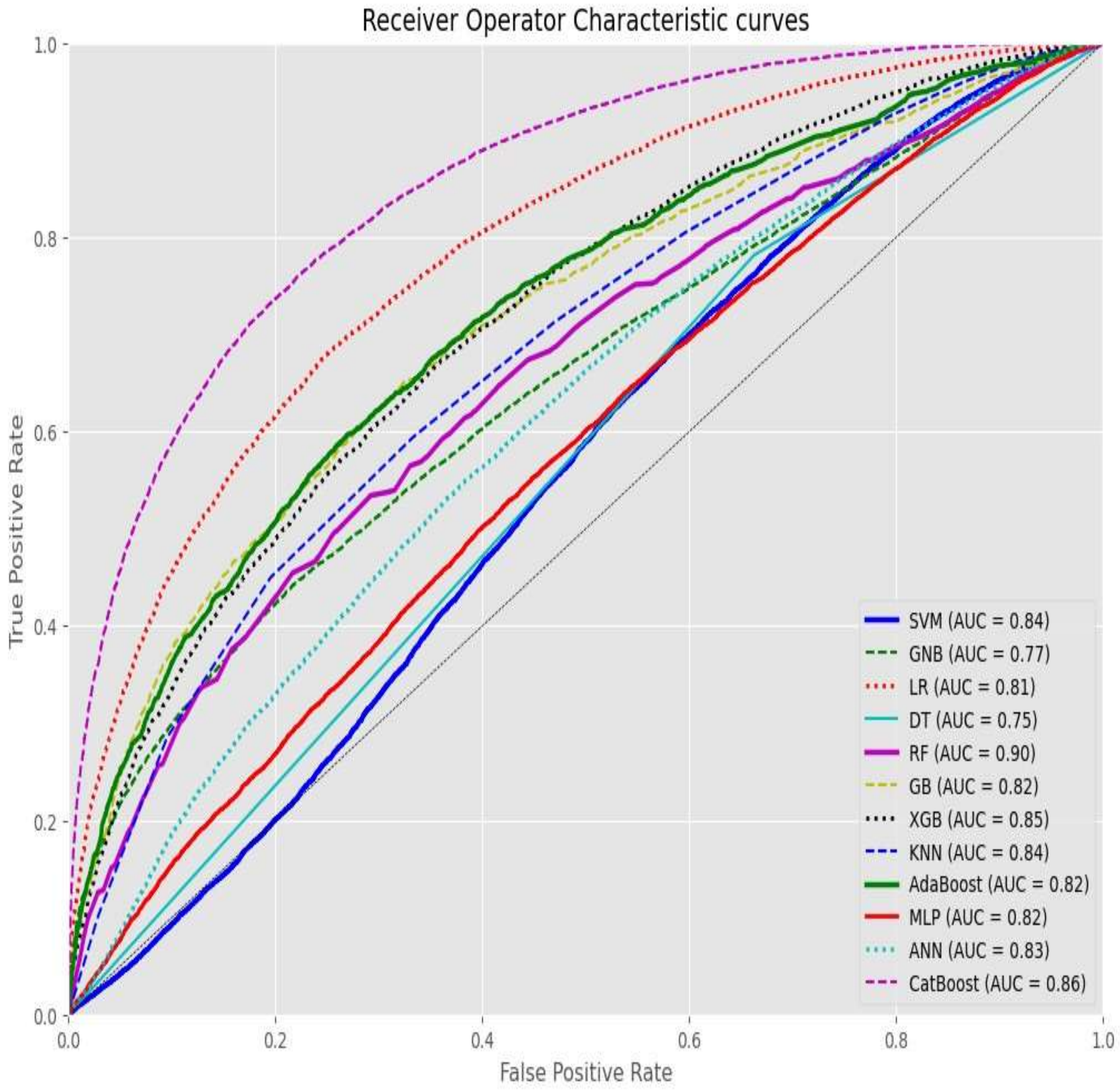

Supplement: Supplementary file 1 [file Data_Sheet_1.PDF]
