# Supplementary material for: Predicting place of delivery choice among childbearing women in East Africa: a comparative analysis of advanced machine learning techniques
Source: Front Public Health. 2024 Nov 27;12:1439320. doi: 10.3389/fpubh.2024.1439320 (PMC11631870; doi:10.3389/fpubh.2024.1439320)

Comprehensive analysis report of performance metrics with Grid search tuning after data were balanced with SMOTE ENN

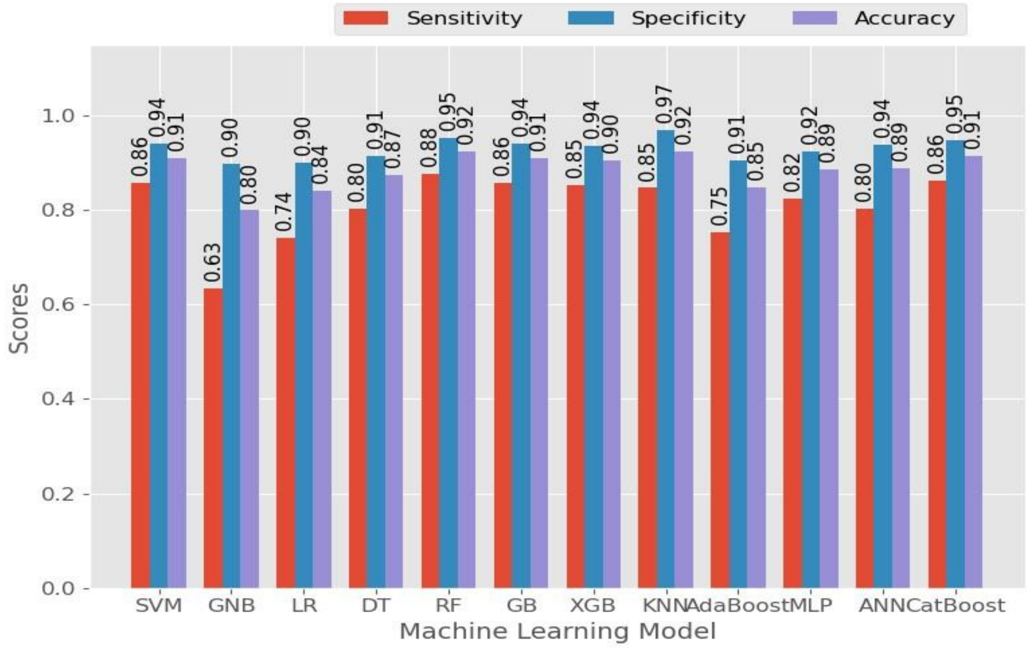

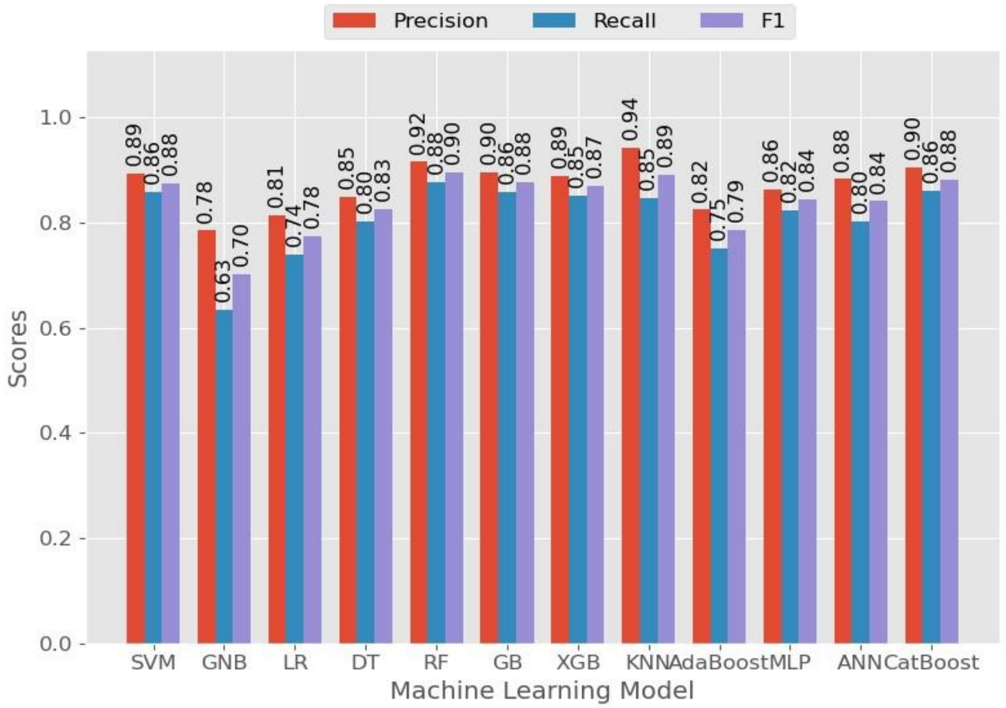

ROC curve using grid search tuning after data were balanced using SMOTE ENN

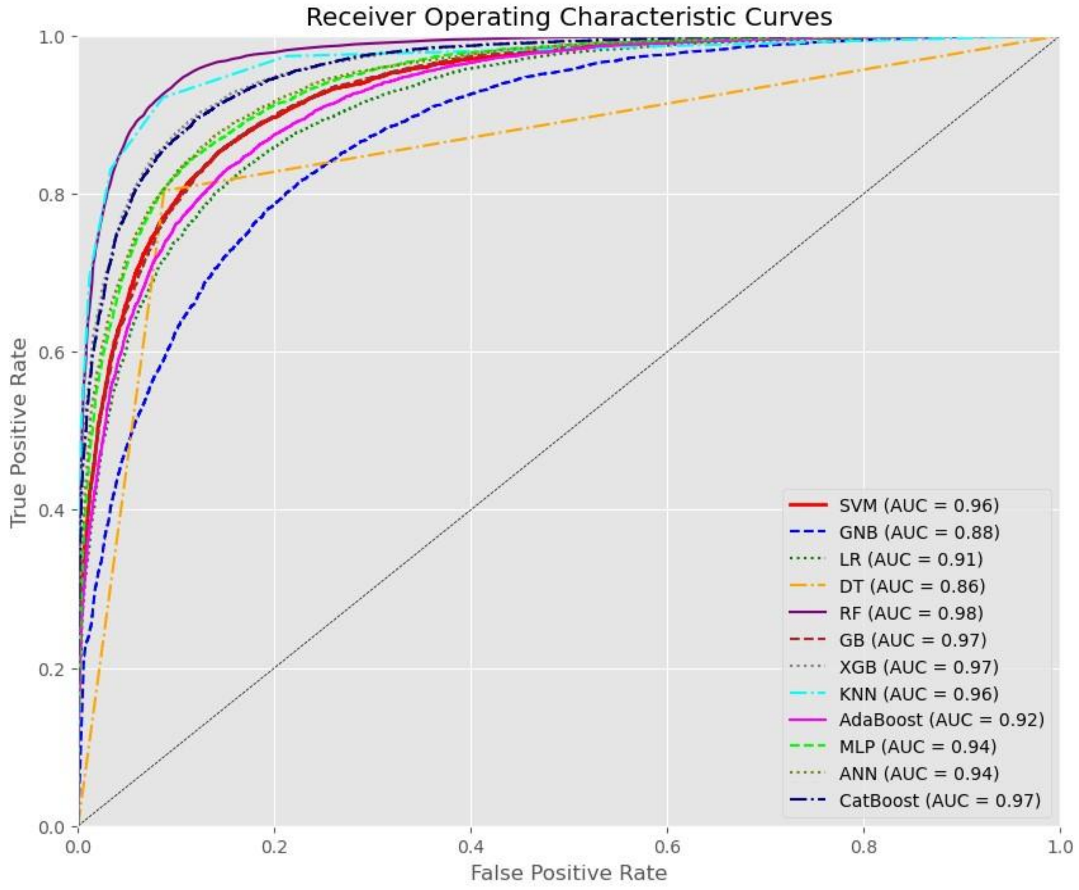

Comprehensive analysis report of performance metrics with Random search tuning after data were balanced with SMOTE ENN

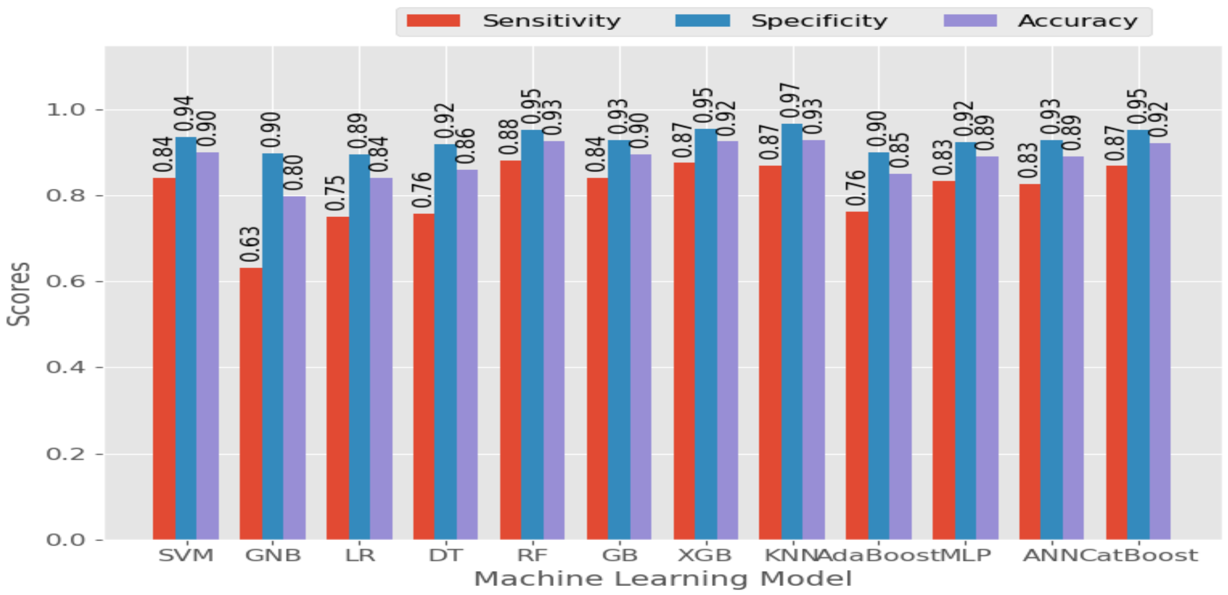

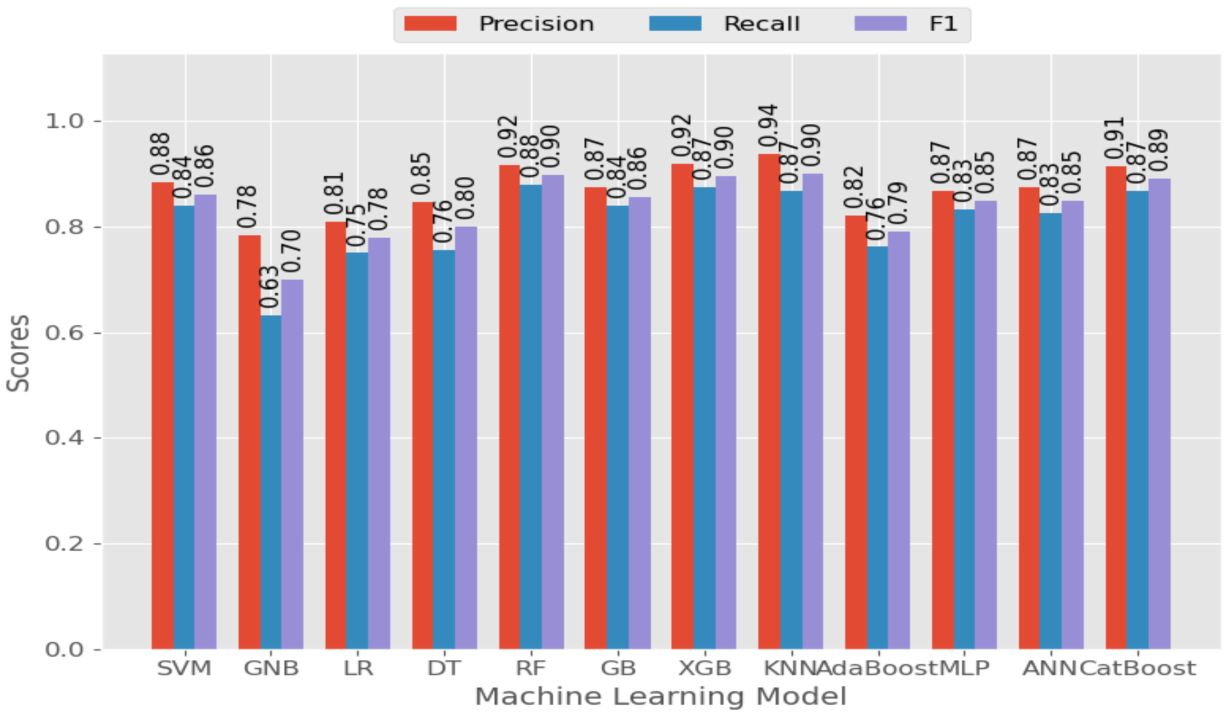

ROC curve using random search tuning after data were balanced using SMOTE ENN

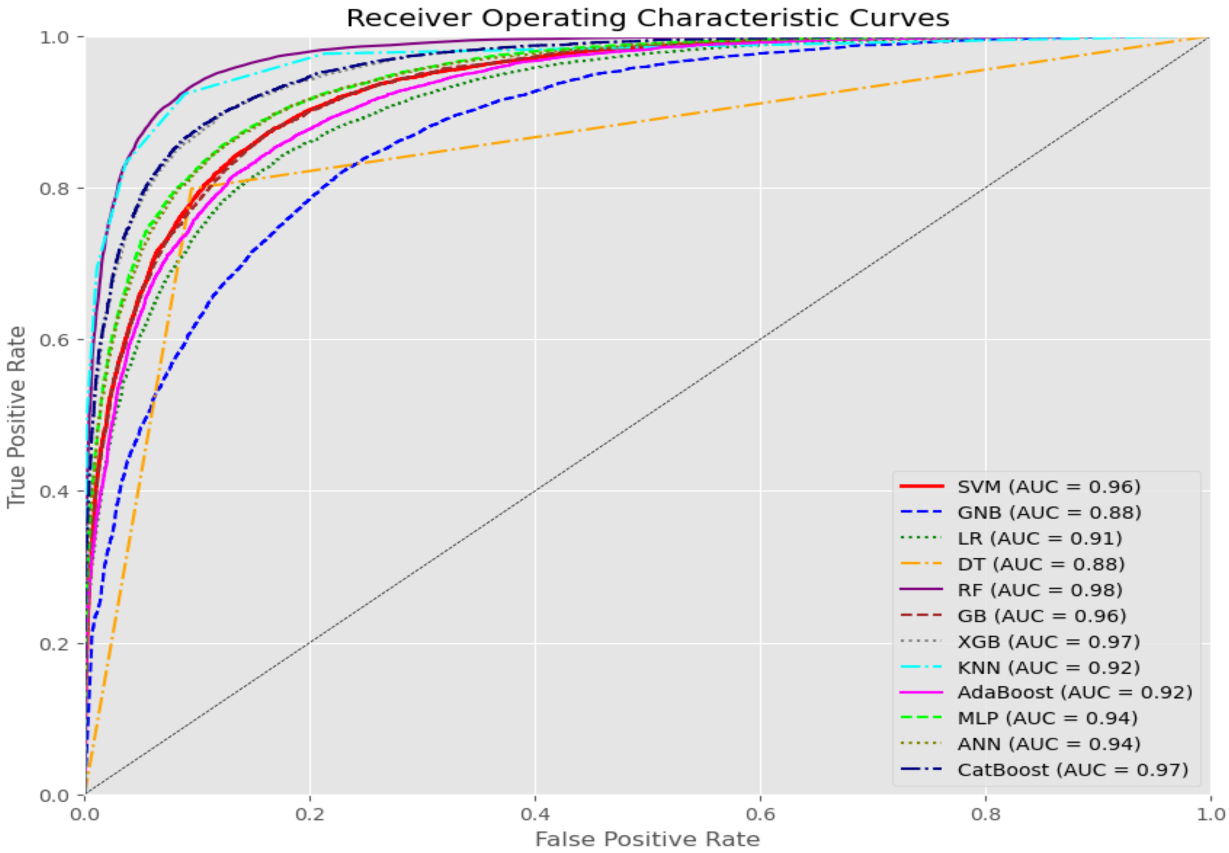

Comprehensive analysis report of performance metrics with Baysian Optimization after data were balanced with SMOTE ENN

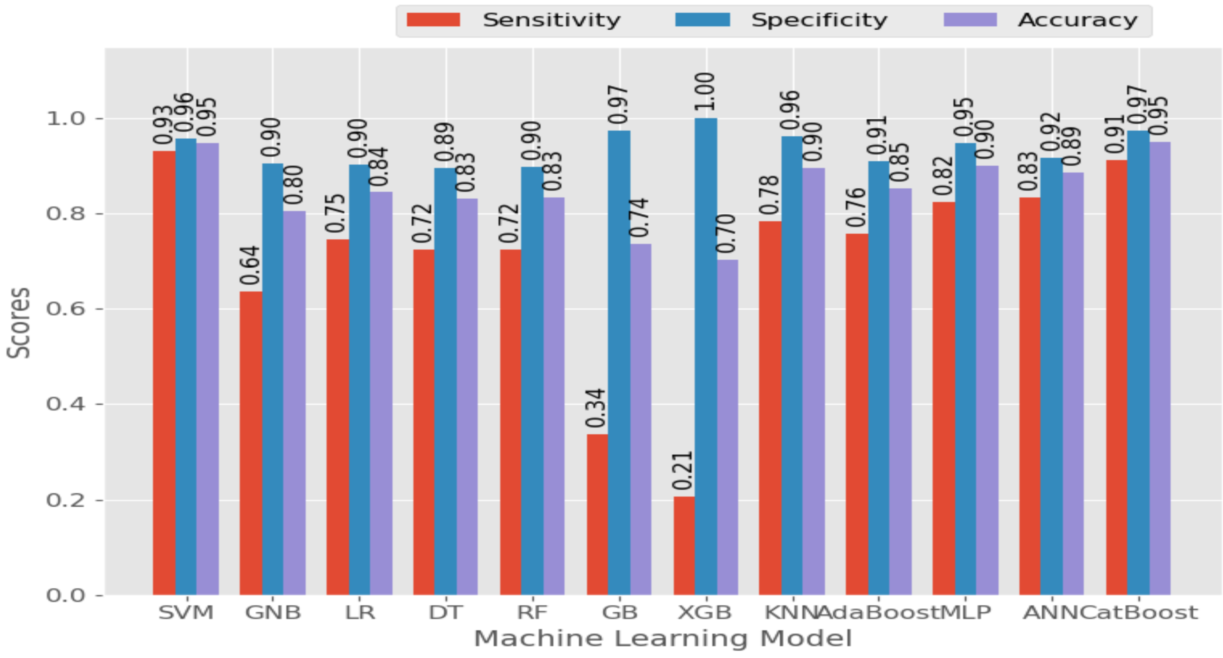

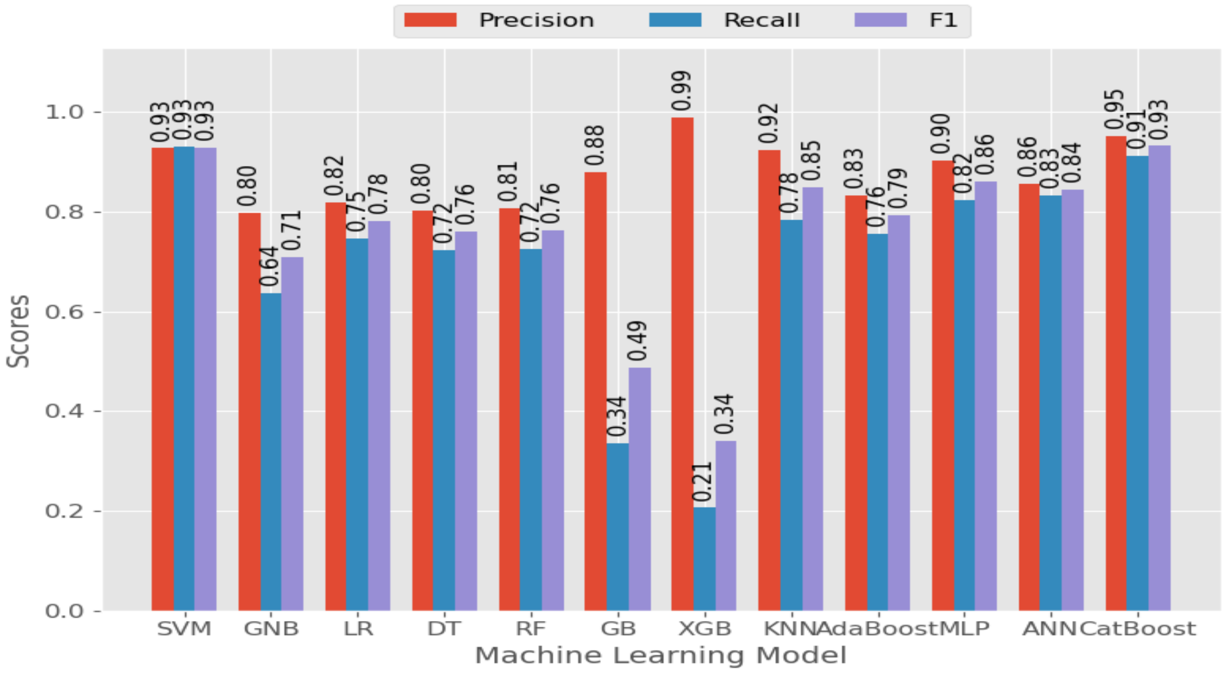

ROC curve using Baysian Optimization tuning after data were balanced using SMOTE ENN

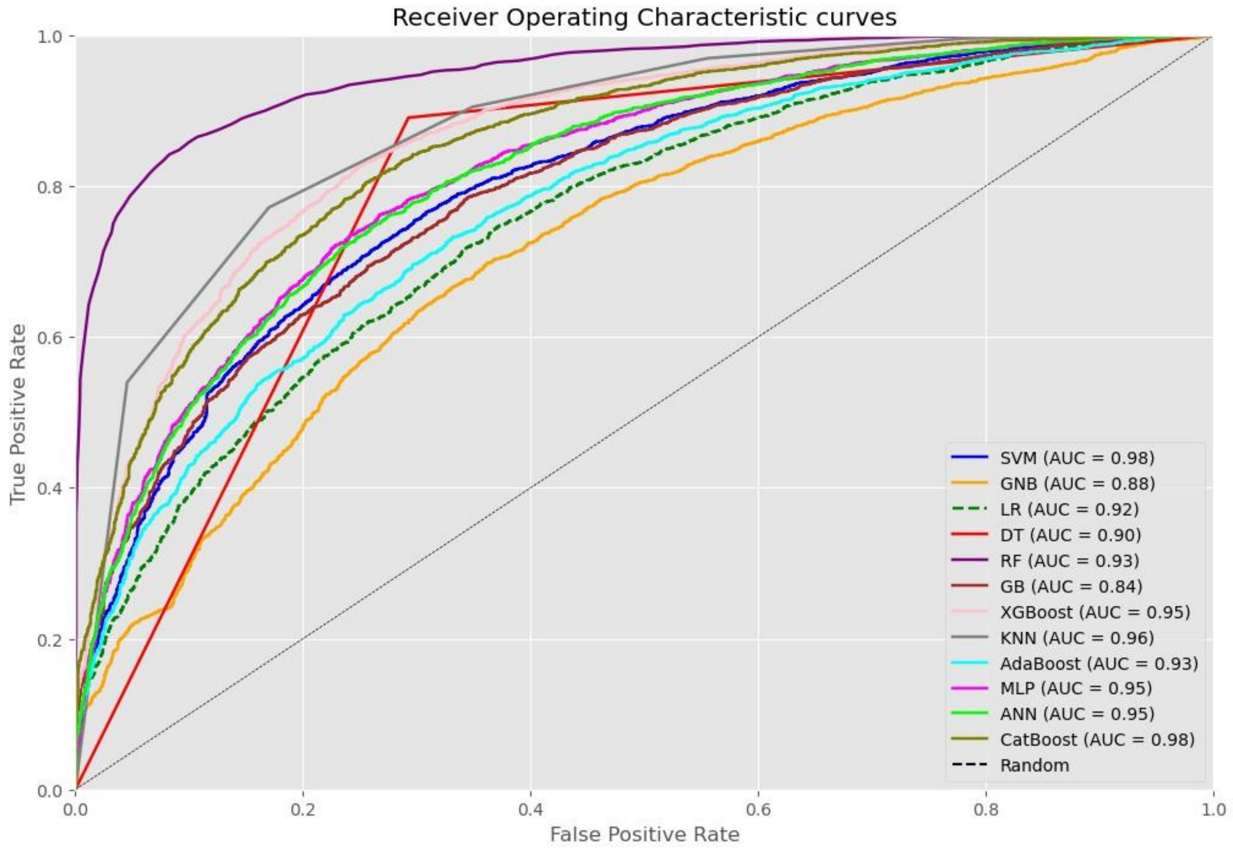

Supplement: Supplementary file 2 [file Data_Sheet_2.PDF]
